# Cellulose-Refined Cholesteric Liquid Crystal Films with Both Right- and Left-Handed Circularly Polarized Light Reflection

**DOI:** 10.1021/acs.biomac.5c01286

**Published:** 2025-12-17

**Authors:** Yu Sotoyama, Yuki Ogiwara, Kazuma Matsumoto, Koya Sunagawa, Naoto Iwata, Seiichi Furumi

**Affiliations:** Department of Chemistry, Graduate School of Science, 26413 Tokyo University of Science, 1-3 Kagurazaka, Shinjuku, Tokyo 162-8601, Japan

## Abstract

Biomass-refinery enables sustainable production of viable fuels and materials. Cellulose has garnered significant attention as the most earth-abundant renewable polymer. Hydroxypropyl cellulose (HPC) derivatives are known to self-organize cholesteric liquid crystal (CLC) mesophase with right-handed macromolecular sense, leading to the chiroptical effect of right-handed circularly polarized light (CPL) reflection. In this report, we establish a promising strategy to prepare solid-state CLC films with both right- and left-handed CPL reflection by shearing chemically modified cellulose derivatives. A key piece of equipment is the rheometer with precisely tunable shear conditions. Both-handed CPL reflection arises from shear-induced enhancement in optical retardation over 300 nm by distorting helical molecular assemblages, as confirmed with the Sénarmont method. The polarization state of incident light is altered within the film thickness direction. Moreover, we successfully fabricate intriguing CLC films with both-handed CPL reflection using inherently right-handed CLCs of the HPC derivative and also inherently left-handed CLCs of the ethyl cellulose derivative.

## Graphical abstract

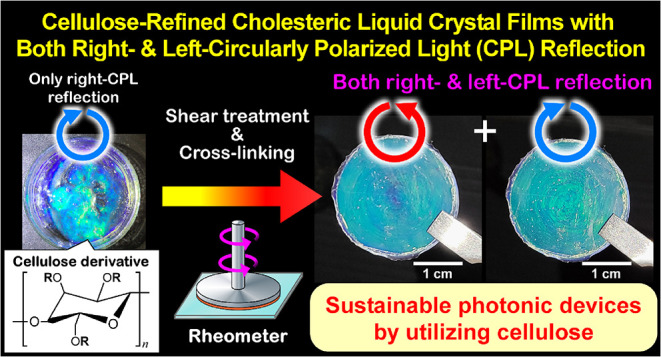

## Introduction

Biomass refinery has been attracting remarkable attention in recent years due to our serious concerns about mass consumption of finite petroleum resources on the planet earth as well as mass production of chemical fuels and products for the modern life.^1–4^ As a result, global warming is progressing by the usage of a terrible amount of petroleum resources, which will continue in the future without taking any action. Moreover, tremendous petroleum-based plastic products made by humans have leaked into various environmental matrices, including the ocean, soil, and atmosphere. Consequently, microplastics are found to be seriously hazardous for the human health.^5^ It would be unavoidable to exhaust fossil resources on the earth if we continue to exploit and consume them. In this context, biomass is one of the environmentally friendly resources derived from living organisms, making its utilization effective to realize a sustainable society, unlike fossil resources. Although it is difficult to immobilize carbon dioxide gas by our artificial technique, the natural events such as photosynthesis by plants or chemosynthesis by microbes facilitate the biochemical absorption and fixation of carbon dioxide gas diffused in the atmosphere, thereby resulting in sustainable production and storage of nonvolatile carbon compounds in the biomass. Therefore, the incineration of biomass-based materials does not change the total amount of carbon atoms in the environment, in principle. This situation would lead to the effective reduction of carbon dioxide emissions, contributing to the prevention of global warming. Currently, many efforts have been made to replace conventional polymers derived from petroleum resources with those derived from biomass.^6,7^

Cellulose is one of the most attractive biomasses that has a low impact on the environment, is safe for human health, and is highly functional as a material.^8–12^ Cellulose is a kind of polysaccharide including glucose as the repeating monomer unit, which is the primary chemical component in the cell walls of plants. Therefore, as another outstanding characteristic, the aid of cellulase enzymes enables on-demand degradation of cellulose in a biological manner, which is especially effective for its derivatives without side chain functionalization or cross-linking.^13–16^ Although cellulose and its derivatives have been utilized as wood, clothing, and paper materials since ancient times, they have recently been re-evaluated not only as environmentally friendly materials but also as functional materials.

For instance, the excellent mechanical toughness or viscoelastic liquid crystallinity are the most fascinating physical properties of cellulose materials.^17–24^

Hydroxypropyl cellulose (HPC), which is one of the cellulose derivatives, has been nowadays applied to pharmaceutical additives and drug delivery materials, arising from its safety for human health and high solubility in both water and organic solvents.^25,26^ Previously, there have been many reports on the appearance of cholesteric liquid crystal (CLC) mesophase by using cellulose materials such as HPC derivatives, cellulose nanocrystals (CNCs), and so forth.^10,17,27–31^ Most interestingly, the esterified HPC derivatives exhibit the CLC phase in both lyotropic and thermotropic manners. In general, CLC materials show the self-organization of helical molecular assemblages of rod-shaped molecules, which cause the periodic modulation of refractive index, thereby leading to the light reflection at an appropriate wavelength region. The reflection peak wavelength (*λ*_ref_) is approximately expressed as the following equation devised by de Vries

1 $$\lambda_{\mathrm{ref}}=n_{\mathrm{av}}\cdot p\cdot \cos\theta$$

where *n*_av_ and *p* represent the average refractive index and helical pitch length of CLC assemblage, respectively. In addition, *θ* stands for the angle between the incident light and the CLC helical axis.^32^ Such a light reflection phenomenon is regarded as a kind of Bragg reflection. Unlike the conventional dielectric multilayered films, selective reflection of circularly polarized light (CPL) is the most important and outstanding property of CLCs. For instance, when unpolarized white light propagates into the right-handed CLC medium, right-handed circularly polarized light (R-CPL) and left-handed circularly polarized light (L-CPL) are reflected and transmitted by the CLC, respectively. Generally, R-CPL is defined when the CPL coming toward the observer is rotated clockwise, and left-handed circularly polarized light (L-CPL) is defined when it is rotated counterclockwise.^33^ In other words, the CPL direction in selective light reflection is the same as the handedness of the CLC helical molecular assemblage. HPC derivatives are commonly known to reflect only R-CPL because they self-organize the right-handed CLC helical molecular assemblages.^29,34^ By utilizing the liquid crystallinity, another important property of CLCs is the on-demand tunability of reflection peak wavelength, that is, reflection color, by solvent addition,^35–37^ temperature,^30,38^ and strain^39,40^ as a result of the geometric changes in CLC helical pitch by the external stimuli. Therefore, there have been hitherto a variety of technological applications to the advanced photonic devices, such as reflective displays,^41,42^ wavelength-tunable lasing devices,^39,43,44^ and stimuli-responsive sensors using the petroleum-refined CLC materials.^45,46^

The current situation seems to result in the research progress of cellulose-based CLC systems being behind that of petroleum-based CLCs. This is because the synthesis and preparation of cellulose-based CLC materials is not straightforward rather than a petroleum-based CLC system due to poor solubility of cellulose materials to solvents as well as restriction of the chemical modification. However, it is of paramount importance to develop photonic devices by using cellulose materials for the realization of a sustainable society. In recent years, there has been an increasing number of reports on the photonic applications such as color inks for three-dimensional printing materials by CNCs as well as chemically modified cellulose derivatives.^47–52^ As mentioned above, the selective CPL reflection is the most unique chiroptical property of the CLCs. Previously, CLC systems that can reflect R-CPL and L-CPL have been presented both theoretically and experimentally.^53,54^ Furthermore, Godinho and colleagues have recently reported an intriguing procedure to control the circular polarization in CPL reflection from the CLC media of CNCs.^55^ Heterogeneous composite CLC films of CNCs combined with a middle layer of nematic liquid crystal (NLC) of 4-butyl-4’-cyanobiphenyl or 4-pentyl-4’-cyanobiphenyl in imitation of the body of the insect *Plusiotis resplendens*,^56^ exhibited both R-CPL and L-CPL reflection due to the large optical anisotropy of the middle NLC layer sandwiched between two CNC films. However, it seems that this fabrication process is complicated and cumbersome, because these composite CLC films are handmade by sandwiching a middle NLC layer between two CLC films of CNCs. Moreover, the effect of birefringence and optical retardation of the middle NLC layer still remains obscure in the experimental evidence, even though both R-CPL and L-CPL reflection phenomenon is generated by the large optical anisotropy of the middle NLC layer.

In this report, we have developed a convenient and promising methodology to generate unique chiroptical properties of both R-CPL and L-CPL reflection from single monolithic CLC films of chemically modified cellulose derivatives fabricated by shear treatment. For this purpose, the key equipment of this study is the rheometer because of its on-demand and fine-tunability of the shear treatment conditions. When the lyotropic CLC fluid mixtures of a cross-linkable HPC derivative with an acrylate monomer were accurately sheared by the rheometer and subsequently irradiated with UV light for the cross-linking reaction, the solid-state CLC films showed both R-CPL and L-CPL reflection by appropriate shear conditions. Importantly, we found that the CPL reflection properties are greatly dependent on the optical retardation of the CLC film, as revealed by the measurement with the Sénarmont method using both a broad-band quarter-wave plate and linear polarizer. The rigorous analysis of optical retardation implied that both R-CPL and L-CPL reflection phenomena can be observed only when the optical retardation exceeds 300 nm, regardless of its reflection peak wavelength. Considering the fact that the CLC films exhibit vivid colors of both R-CPL and R-CPL reflection as well as an increase in optical retardation, it is plausible that shear treatment at the appropriate conditions gives rise to the formation of heterogeneous CLC films with two regions of different molecular orientation in the thickness direction of a single monolithic film. One is the region of well-ordered CLC helical molecular assemblage near the outermost surfaces of the film induced by the anchoring effect, and the other is that of disturbed helical molecular structure around the middle layer inside the film induced by mechanical shear force. In this way, the single monolithic CLC films with a gradient heterogeneous structure by shear treatment might be similar to the handmade composite CLC films sandwiched with a middle NLC layer, as reported by Godinho and colleagues.^55^ From a technological viewpoint, it would be greatly advantageous to fabricate single monolithic CLC films by shear treatment in one step. In this way, the molecular orientation state and optical retardation of CLC films could be finely controlled by shear treatment with the rheometer. This report paves the way for not only the general preparation of single monolithic CLC films with both R-CPL and L-CPL reflection phenomena by shear treatment, but also the sustainable photonic applications by refining cellulose.

## Experimental Section

### Synthesis and Characterization of a Cross-Linkable HPC Derivative

The materials, synthesis procedure, and characterization of a cross-linkable HPC derivative are detailed in the Supporting Information. Briefly, hydroxy groups of a pristine HPC were chemically modified not only by carbamation with 2-acryloyloxyethyl isocyanate but also by esterification with acetyl chloride to yield the HPC derivative possessing acryloyl side chains as cross-linkable units (Figure 1, i). The crude product was elaborately purified by four rounds of reprecipitation from acetone to ultrapure water. The dried HPC derivative was analyzed by both FT-IR and ^1^H NMR spectral measurements. The experimental results and analytical procedures are presented in the Supporting Information.

**Figure 1:**
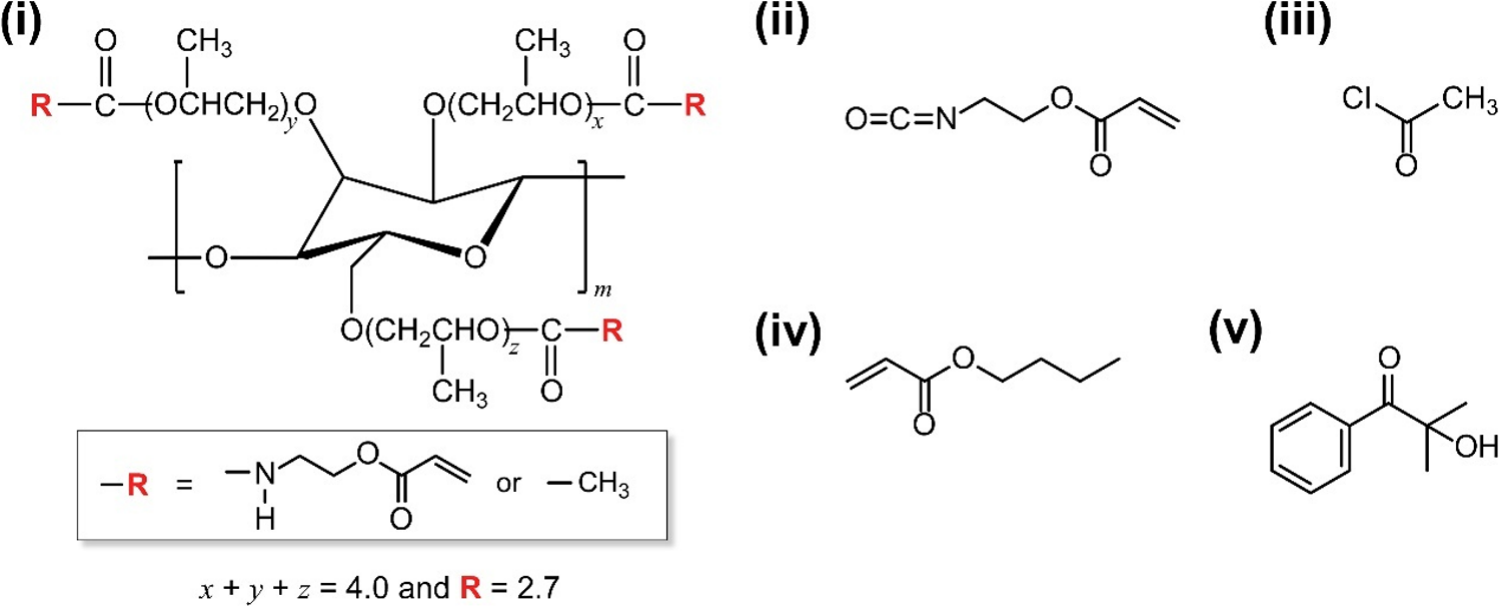
Chemical structures of a cross-linkable HPC derivative (i) and reagents (ii–v) used in this study. (i) An HPC derivative possessing both 2-acryloyloxyethyl carbamoyl and propionyl groups in the side chains. (ii) 2-Acryloyloxyethyl isocyanate adopted for carbamation. (iii) Acetyl chloride for esterification. (iv) Butyl acrylate as a solvent of lyotropic CLC. (v) 2-Hydroxy-2-methylpropiophenone as a photoradical initiator.

### Fabrication Procedure of CLC Films by Shear Treatment with the Rheometer

First, lyotropic CLC mixtures were prepared by dissolving the cross-linkable HPC derivative in a mixture of butyl acrylate (Figure 1, iv) and 2-hydroxy-2-methylpropiophenone (Figure 1, v) at certain concentrations. The reflection peak wavelength of lyotropic CLC was easily adjusted by changing the concentration of the HPC derivative, as given in Table 1. Successively, the lyotropic CLC mixtures were sheared by the stress-controlled rheometer (Anton Paar, MCR 102), which allowed precise adjustment of the shear treatment conditions. In the experiment, most of the shear treatments were carried out at a shear rate at the outermost positions of the upper jig (*γ̇*_out_) of 0.5 s^–1^ (Figure 2, *b*). After shear treatment of the lyotropic CLC mixtures, *γ̇*_out_ was immediately set to 0 s^–1^ (Figure 2, *c*). At this stage, the lyotropic CLC mixtures were allowed to be static for a certain period of time. Finally, the solid-state CLC films were prepared by a photoinduced cross-linking reaction of the HPC derivative and butyl acrylate (Figure 2, *d*). Details of the fabrication procedure of lyotropic CLC mixtures and CLC films are explained in the Supporting Information.

**Table 1:** Experimental Conditions for Fabrication of the CLC Films without and with Shear Treatment

sample code	HPC derivative conc. (wt %)^a^	condition of shear treatment		λ_ref_ (nm)^c^
		*d*_set_ (mm)^b^	recovering time (s)	
**Film a**	77.0	no shear treatment		∼469
**Film b**	72.9	no shear treatment		∼520
**Film c**	71.5	no shear treatment		∼612
**Film 1**	77.0	0.50	200	∼453
**Film 2**	72.9	0.50	200	∼530
**Film 3**	72.7	0.50	200	∼580
**Film 4**	74.4	0.80	200	∼549
**Film 5**	74.4	0.30	200	∼539
**Film 6**	71.9	0.80	200	∼609
**Film 7**	71.9	0.30	200	∼593
**Film 8**	77.0	0.80	200	∼408
**Film 9**	77.0	0.50	200	∼406
**Film 10**	77.0	0.27	200	∼409
**Film 11**	77.0	0.21	200	∼407
**Film 12**	77.0	0.15	200	∼422
**Film 12’** ^d^	67.9	no shear treatment		∼506
**Film 13**	77.0	0.10	200	∼419
**Film 14**	78.2	0.50	10	∼448
**Film 15**	77.8	0.50	100	∼451
**Film 16**	77.8	0.50	1200	∼453

a Weight concentration of the HPC derivative in lyotropic CLC.

b Geometric gap distance between the upper and lower jigs set by the rheometer.

c Average reflection wavelength calculated from the transmission spectra of CLC films when irradiated with either left or right circularly polarized light in radial direction.

d **Film 12’** was prepared by the cross-linkable HPC derivative with *AcC* of ∼0.02 and *EtE* of ∼2.90. In general, for the HPC derivatives with higher substitution degrees, that is, the sum of *AcC* and *EtE*, the reflection peak shifts toward the shorter wavelength side. Consequently, the concentration of the cross-linkable HPC derivative in butyl acrylate for **Film 12’** was lower than those in the other films.

**Figure 2:**
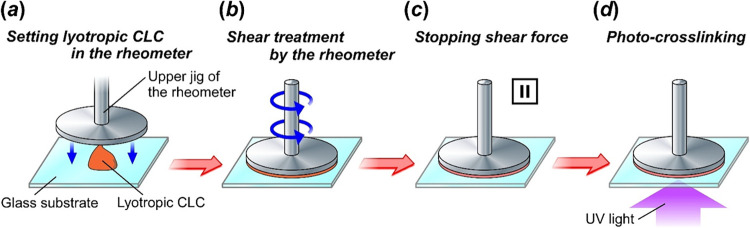
Preparation procedure of the solid-state CLC films of HPC derivative and butyl acrylate that reflect both right- and left-handed circularly polarized light. (*a*) Settling the lyotropic CLC fluid mixture on the sample stage of the rheometer. (*b*) Shear treatment by the jig of rheometer. (*c*) Stopping the shear force to recover helical molecular orientation of the lyotropic CLC. (*d*) Irradiation with UV light at 365 nm for the cross-linking reaction of the lyotropic CLC mixture to form the solid-state CLC film.

### Optical Measurements and Analysis of CLC Films

Optical measurements and analysis of CLC films are of paramount importance in this study. Therefore, the details are explained as follows. The reflection images of right-handed circularly polarized light (R-CPL) and left-handed circularly polarized light (L-CPL) were taken by using a camera (Apple, iPhone 13). Unpolarized white light was irradiated onto the CLC films, and its reflection light was taken through a quarter-wave plate and a linear analyzer. By setting the direction of the optical axis of the quarter-wave plate and the polarization axis of the linear analyzer to ±45°, we obtained R-CPL and L-CPL reflection images. To qualitatively evaluate the optical retardation (*R*_e_) and to determine the optical axis of the CLC films, the transmission images of the CLC film under crossed-Nicols were also recorded.

The reflection properties of CLC films were evaluated from the transmission spectra when the samples were irradiated with white circularly polarized light (CPL). This is because the decline of the transmittance is due to the reflection of light by the CLC film. The average reflection properties over the entire film thickness direction are measured by transmission spectra, while the reflection spectral measurements provide information of only the light reflected at the film surface. An original microscopic spectroscopy system was arranged to measure the R-CPL and L-CPL transmission spectra (Supporting Information, Figure S1A).^57^ White light from a halogen light source (Olympus, TH4-100) was reflected by a mirror and focused by a long working objective lens for microscopy (Olympus, SLMPLN20X; working distance: 25 mm) onto the sample. In this measurement system, the incident light was focused to a size of ∼1 mm in diameter. The light emitted from the objective lens was converted to L-CPL or R-CPL by passing it through both a linear polarizer (Opto-Line Inc., SPF15-D50) and a quarter-wave film (MeCan Imaging Inc., HCR140N) attached to the glass surface. The handedness of the CPL was controlled by setting the direction of the polarization axis of the linear polarizer and the fast axis of the quarter-wave film to be ±45°. The transmission light of the sample was focused by doublet achromatic lenses and then guided by an optical fiber to a charge-coupled-device (CCD) spectrometer (Ocean Optics, HR4000) to measure their spectra. This optical system herein enabled measurement of spectra in the full visible wavelength range of 400–750 nm. By placing the sample on an XYZ stage, the reflection peak wavelength (*λ*_ref_) of the CLC film at different positions, that is, different *r*, was determined. The *λ*_ref_ was defined as the average of the minimum transmittance wavelength in the transmission spectrum.

The degree of molecular orientation induced by shear treatment was investigated by measuring *R*_e_ and birefringence (Δ*n*) using the Sénarmont method.^58^ The measurement system for the Sénarmont method is illustrated in Figure S1B of the Supporting Information. White light emitted from a halogen light source (Olympus, TH4-100) was converted to linearly polarized light by a linear polarizer (Opto-Line Inc., SPF15-D50), then focused by a long working objective lens for a microscope (Olympus, SLMPLN20X; working distance: 25 mm), and irradiated onto the CLC film. The CLC film was set so that the angle between the polarization axis of the linear polarizer and the optical axis of the sample was 45°. The direction of the optical axis of the sample was determined before the measurement by observing the transmission image under crossed-Nicols. After the transmission light was focused with doublet achromatic lenses, the transmission spectrum was measured with the CCD spectrometer (Ocean Optics, HR4000) through a broad-band quarter-wave plate (Sigma Koki Co., Ltd., WPQW-VIS-4M) and a linear analyzer (Sigma Koki Co., Ltd., SPF-50C-32). The quarter-wave plate was set so that the direction of its fast axis was parallel to the polarizer, and the analyzer was set so that its polarized axis was orthogonal to the polarizer. Notice that only the direction of the axis of the detector is changed in the measurement, while the direction of the axis of the polarizer and the quarter-wave plate is maintained. A dark reference measurement was performed with the polarizer and the analyzer in crossed-Nicols, and a reference measurement in light was performed with the polarization axis of the analyzer set to be parallel to that of the polarizer. *R*_e_ and Δ*n* were calculated from the rotation angle (*α*°) of the analyzer at the arbitrarily set wavelength (*λ*_set_) until the transmission light intensity was at its lowest, using eqs 2, 3, and 4. In this study, *δ* is the phase difference in the unit of radians and *d*_act._ is the actual film thickness. Because the *d*_act._ of each film is the actual thickness measured with a micrometer gauge, there was a slight difference between *d*_set_ and *d*_act._.

2 δ=2α×π/180

3 $$R_{e}=\frac{\delta \lambda_{\mathrm{set}}}{2\pi }$$

The Sénarmont method could not distinguish *R*_e_ corresponding to a phase difference of even multiples of π due to its measurement principle. Therefore, to confirm that the *R*_e_ calculated using the Sénarmont method is appropriate, we also measured the transmission spectra under crossed-Nicols. For the measurement of this spectrum, a system in which the quarter-wave plate was excluded from the measurement system shown in Figure S1B of the Supporting Information, and the polarizer and analyzer were set orthogonally to each other, was used. We confirmed that *R*_e_ is an appropriate value by checking that the measured transmission spectrum agreed with the theoretical spectrum calculated from eq 4, where *τ*_⊥_(*λ*) is the transmittance, *R*_e_ is the measured value from the Sénarmont method, and *θ* is the angle between the polarization axis of the detector and the optical axis of the sample, which in this study was set to be *θ* = 45°.

4 $$\tau_{⊥}\left(\lambda \right)=\sin^{2}2\theta \cdot \sin^{2}\frac{\pi R_{e}}{\lambda }$$

For instance, the transmission spectra of **Film 1** under crossed-Nicols and the theoretical transmission spectra calculated using eq 4 and the measured *R*_e_ are shown in Figure S2 of the Supporting Information. Both transmission spectra corresponded, which means that the measured *R*_e_ value is valid.

## Results and Discussion

### Synthesis and Characterization of a Cross-Linkable HPC Derivative

In our previous report, photopolymerization of the lyotropic CLCs of the HPC derivative without cross-linking groups in the side chains and 4-hydroxybutyl acrylate gave rise to the phase separation between the HPC derivative and poly­(4-hydroxybutyl acrylate). Consequently, the reflection peak completely disappeared after the polymerization.^37^ From our investigation, we designed and synthesized a cross-linkable HPC derivative possessing 2-acryloyloxyethyl carbamoyl and acetyl groups in its side chains in order to avoid phase separation after cross-linking reaction (Figure 1, i). The hydroxy groups of a pristine HPC were chemically reacted by carbamation and esterification of with 2-acryloyloxyethyl isocyanate (Figure 1, ii) and acetyl chloride (Figure 1, iii), respectively, according to our previously reported procedure with a slight modification.^34^ The detailed synthesis procedure of the HPC derivative is also described in the Supporting Information.

As-synthesized HPC derivative was characterized by both FT-IR and ^1^H NMR spectroscopic measurements according to the previous studies.^59^ The experimental results are shown in Figure S3 of the Supporting Information. In the FT-IR spectrum, this cross-linkable HPC derivative exhibited the disappearance of a broad band around 3100–3600 cm^–1^ arising from the O–H stretching vibration of hydroxyl groups of pristine HPC.^59^ Concomitantly, a sharp peak originating from the C=O stretching vibration appeared at ∼1700 cm^–1^ for the HPC derivative (Supporting Information, Figure S3A). These results suggest that the hydroxyl groups of the side chains of HPC are carbamated by 2-acryloyloxyethyl isocyanate or esterified by acetyl chloride. The ^1^H NMR spectra of the HPC derivative were measured to stoichiometrically evaluate the substitution degrees of the hydroxyl groups of HPC with 2-acryloyloxyethyl isocyanate and acryloyl chloride (Supporting Information, Figure S3B).^34^ Hereafter, the average number of hydroxyl groups in a monomer unit of HPC carbamated by 2-acryloyloxyethyl isocyanate is defined as *AcC*, and that esterified by acetyl chloride is defined as *EtE*. Since the HPC monomer unit has three hydroxyl groups, the sum of *AcC* and *EtE* should be 3.00 when pristine HPC is completely carbamated or esterified. The *AcC* and *EtE* values were numerically calculated from the integrated values of the peaks in the ^1^H NMR spectrum of the HPC derivative by using eqs 5 and 6.

5 $$\frac{a}{W}=\frac{3\mathrm{EtE}}{7+6\mathrm{MS}+7\mathrm{AcC}+3\mathrm{EtE}}$$

6 $$\frac{b}{W}=\frac{\mathrm{AcC}+\mathrm{EtE}}{7+6\mathrm{MS}+7\mathrm{AcC}+3\mathrm{EtE}}$$

where *a* stands for the integrated value of “peak a” at ∼2.0 ppm, attributed to the protons of the methyl group of the acetyl group, *b* is the integration value of “peak b” at ∼5.0 ppm, attributed to the protons of the methine group of the carbamated or acetylated hydroxypropyl group, *W* is the sum of the integrated values of all peaks assigned to the protons of the HPC derivative, and *MS* is the molar substation degree of pristine HPC. The mathematical derivation of eqs 5 and 6 is available in the Supporting Information. It should be noted that the *MS* value is determined to be 4.00, as also described in the Supporting Information. The cross-linkable HPC derivative with *AcC* of ∼0.02 and *EtE* of ∼2.68 was used in almost all of the experiments of this study.

### CLC Films of HPC Derivative Fabricated without Shear Treatment

In order to fabricate the solid-state CLC films without and with shear treatment, we prepared lyotropic CLC fluid mixtures, including the cross-linkable HPC derivative as a base CLC material (Figure 1, i), butyl acrylate as a solvent of lyotropic CLC (Figure 1, iv), and 2-hydroxy-2-methylpropiophenone as a photoradical initiator (Figure 1, v). At this time, the concentration of HPC derivative in butyl acrylate was changed in the range of 72.0–78.0 wt % to tune the reflection peak wavelength, while that of 2-hydroxy-2-methylpropiophenone was standardized at ∼0.7 wt %. The mixtures exhibited a lyotropic CLC phase with visible reflection at room temperature, and the reflection peak wavelength shifted to the longer wavelength side with a decrease in the concentration of HPC derivative (Supporting Information, Figure S4). This is because the *p* value of lyotropic CLC phase formed by the HPC derivative enlarges upon adding butyl acrylate in the similar way as the previous report on aqueous solutions of pristine HPC by Gray and a co-worker.^35^

First, we fabricated the CLC films without shear treatment as references and compared the results of the CLC films with shear treatment. In this study, three kinds of CLC films with a reflection peak approximately at 470, 520, and 620 nm were prepared from the lyotropic CLC mixtures with the concentrations of the cross-linkable HPC derivative at 77.0, 72.9, and 71.5 wt %, respectively, as shown in Table 1. The lyotropic CLC fluids were sandwiched between a pair of glass substrates with a gap of ∼0.5 mm adjusted by polytetrafluoroethylene film spacers and subsequently irradiated with UV light at 365 nm to generate the cross-linking reaction between acryloyl groups of HPC derivative and butyl acrylate (Table 1, **Films a**–**c**). At this time, the intensity of UV light and irradiation time were set to 80 mW/cm^2^ and 360 s, respectively. Although the glass substrates used in this study exhibited optical transparency of 80% or more at the irradiation wavelength of 365 nm, this irradiation procedure with UV light was sufficient to obtain the cross-linked CLC films. Indeed, the fluidity of lyotropic CLC mixtures vanished, whereupon the resultant CLC films exhibited durably robust solid-state with stable reflection colors.

In a preliminary experiment, we measured the transmission spectra of **Films a–c** using unpolarized white light for probing. The reflection peak appeared at ∼465 nm for **Film a**, ∼510 nm for **Film b**, and ∼610 nm for **Film c** (Supporting Information, Figure S5). Notably, the reflection peak wavelengths of the CLC films and lyotropic CLC fluids were almost identical. In this way, the helical molecular assemblages of lyotropic CLCs could be thoroughly maintained in solid-state CLC films by the cross-linking of HPC derivatives with butyl acrylate.

Successively, the chiroptical properties of the CLC films were evaluated by transmission spectral measurement with L-CPL or R-CPL as a probing light. Figure 3 shows the transmission spectra of L-CPL and R-CPL of the CLC films without shear treatment (**Films a–c**). As evident from Figures 3A and 3B, the circularly polarized transmission spectroscopic shape was drastically altered by switching the probing light between L-CPL and R-CPL. In the L-CPL transmission spectra of **Films a–c**, a broad peak was observed at ∼469 nm for **Film a**, ∼520 nm for **Film b**, and ∼612 nm for **Film c** (Figure 3A). The decrease in transmittance might be ascribed to the reflection of L-CPL. However, their peak intensity was quite low, and the reflection color of each film was opaque (Figure 3A, insets). On the other hand, sharp peaks appeared in the R-CPL transmission spectra of **Films a–c** (Figure 3B). For example, the transmittance decreased by ∼70% at 471 nm in the case of **Film a** (Figure 3B, light gray line). In addition, the R-CPL reflection image of **Film a** exhibited a vivid blue color (Figure 3B, bottom inset). Such a reflection of R-CPL was also observed for **Film b** and **Film c** by the CLC helical molecular assemblage of HPC derivative with butyl acrylate, except for the difference in the reflection peak wavelength and reflection color. According to the previous reports, it is known that the CLC materials of HPC derivatives generally show predominant R-CPL reflection because they intrinsically form a right-handed helical molecular assemblage.^29,34^ However, **Films a–c** exhibited slight reflection properties of L-CPL even though their reflection intensity is very weak. In the precedents by Takezoe and co-workers, they observed the reflection of L-CPL from the right-handed CLC materials when the R-CPL was incident at an oblique angle of ∼46° to the helical molecular axis of the CLC medium.^60,61^ From this fact, it is suggested that the helical axes of **Films a–c** are not perfectly parallel to the film thickness direction due to the lack of shear treatment. Therefore, the chiroptical property of CPL reflection might be greatly dependent on the orientation state of the CLC helical molecular assemblage. Moreover, Tokita and colleagues adopted the rheometer to apply steady shear or large amplitude oscillatory shear to a smectic liquid crystalline polymer, which enabled the on-demand control of its orientation state.^62^ This precedent and our serendipitous finding on the L-CPL reflection by CLC films of HPC derivatives motivated us to establish a promising methodology to prepare unique CLC films with both R-CPL and L-CPL reflection by changing the orientation state of CLC helical molecular assemblage by applying the mechanical shear force under appropriate conditions with the rheometer.

**Figure 3:**
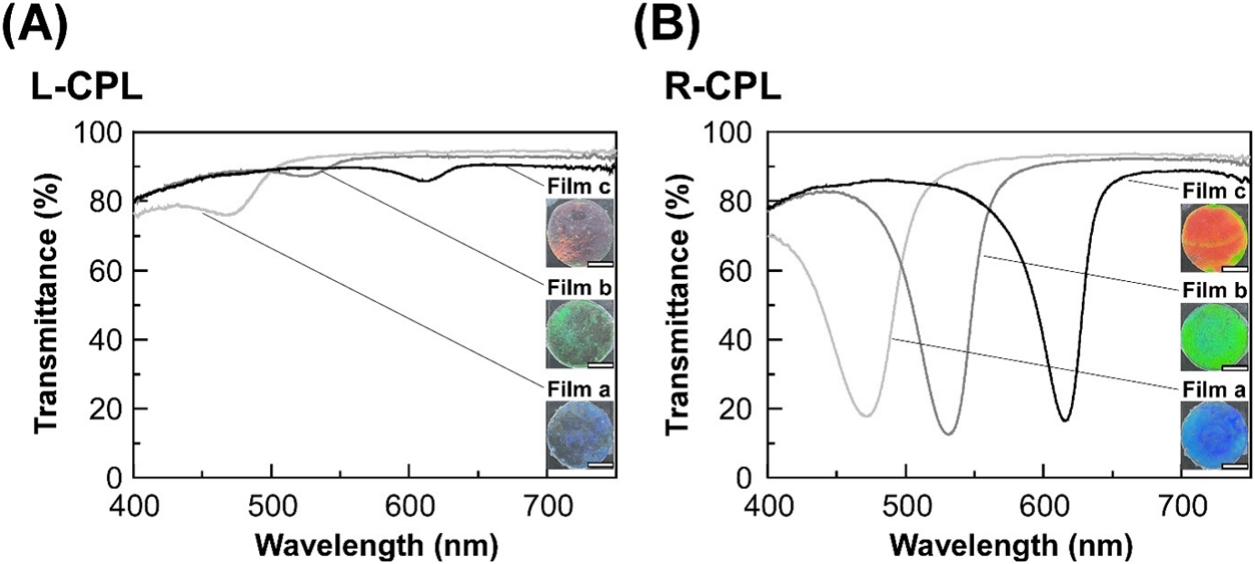
L-CPL (A) and R-CPL (B) transmission spectra of **Film a** (light gray lines), **Film b** (dark gray lines), and **Film c** (black lines), which were fabricated from lyotropic CLC mixtures of the HPC derivative at its concentrations of 77.0, 72.9, and 71.5 wt %, respectively, without shear treatment by the rheometer (Table 1). Insets show the reflection images of L-CPL (panel A, insets) and R-CPL (panel B, insets) observed through both quarter-wave plate and linear polarizer. White scale bars in the images denote 10 mm.

### CLC Films of HPC Derivative Fabricated by Shear Treatment with the Rheometer

As motivated by the preceding section, we prepared the CLC films from a lyotropic CLC fluid mixture of the cross-linkable HPC derivatives with butyl acrylate by shear treatment with the rheometer. Figure 3 depicts the preparation steps of the CLC films by shearing the lyotropic CLCs with the rheometer. First, the lyotropic CLC fluid was settled on the sample stage of the rheometer equipped with a parallel plate jig with 25 mm diameter (Figure 2, *a*), and the upper jig was lowered to flatten the CLC to a value of *d*_set_, where *d*_set_ means a geometric gap distance between the upper and the lower jigs of the rheometer. Second, the shear treatment was performed by rotating the upper jig at a constant shear rate for 300 s (Figure 2, *b*). It should be noted that the parallel plate jig provides a distribution of shear rate (*γ̇*) along the direction of the radius (*r*) (Supporting Information, Figure S6). Therefore, the *γ̇* at the positions of *r* is accurately calculated as the following eq 7.

7 $$\mathrm{\gamma ̇}=\frac{r}{12.5}\times {\mathrm{\gamma ̇}}_{\mathrm{out}}$$

where *γ̇*_out_ is defined as the shear rate at the outermost positions of the upper jig, that is, γ̇ at the positions of *r* = 12.5 mm. After the shear treatment, *γ̇*_out_ was immediately set to 0 s^–1^ to settle the lyotropic CLC for 2–3 min (Figure 2, *c*). In this study, the time that was kept static is defined as the recovering time. Finally, the solid-state CLC films were immobilized by irradiation of UV light of 365 nm at 80 mW/cm^2^ for 360 s for the cross-linking reaction of acryloyl groups of the HPC derivative and butyl acrylate (Figure 2, *d*). The fabrication conditions of solid-state CLC films by shearing with the rheometer are listed in Table 1.

In a preliminary experiment of shear treatment by the rheometer, we fabricated a CLC film using a lyotropic CLC mixture with the polymer concentration of 78.2 wt %. At this time, the shear treatment was carried out at *γ̇*_out_ = 5.0 s^–1^. The L-CPL and R-CPL transmission spectra of the film at *r* = 0, 3, 6, and 9 mm are shown in Figure S7 of the Supporting Information. The reflection peak was clearly observed for the L-CPL transmission spectra at *r* = 0 and 3 mm. However, for those at *r* = 6 or 9 mm, we observed a very broad band with a weak intensity. The results suggested that L-CPL is hardly reflected in the outer region of the film at *r* > 6 mm (Figure S7A). As expected, a sharp peak was observed in the R-CPL transmission spectra at *r* = 6 or 9 mm, while this peak became broad when measured at *r* = 0 or 3 mm (Figure S7B). Therefore, it was found that the CPL reflection properties of this CLC film are largely dependent on the *r* value. In the outer region of the film at *r* > 6 mm, the R-CPL reflection was dominant, because no intense peak was observed for the L-CPL transmission spectra. Conversely, on the inner side of the film at *r* < 3 mm, it turned out that the CLC film can reflect both L-CPL and R-CPL because the reflection peak wavelength and spectral shape were almost identical for each spectrum. Such different CPL reflection properties depending on *r* can be ascribed to the different orientation states of the CLC helical molecular assemblage in the radial direction. This is reasonable because there is a distribution in the shear rate along the radius during the shear treatment, as expressed in eq 7. From the overall results, we considered that the mechanical shear treatment at slower shear rates, such as *γ̇*_out_ < 5.0 s^–1^, would enable the preparation of CLC films that can reflect both L-CPL and R-CPL uniformly in the radial direction. Moreover, the previous report has also shown that the orientation history induced by the shear treatment at higher share rate is more likely to disappear quickly.^63^ Therefore, we attempted to fabricate the CLC films with both L-CPL and R-CPL reflection by reducing the *γ̇*_out_ value from 5.0 to 0.5 s^–1^ in the shear treatment with the rheometer.

After that, we fabricated **Film 1** by the shear treatment of *γ̇*_out_ = 0.5 s^–1^ and *d*_set_ = 0.50 mm on the lyotropic CLC mixture of the HPC derivative at its concentration of 77.0 wt %, followed by irradiation with UV light. The L-CPL and R-CPL transmission spectra of **Film 1** are shown in Figure 4A. Interestingly, we observed both L-CPL and R-CPL reflection phenomena from **Film 1** at ∼455 nm (Figure 4A, left and right panels, dotted black lines). In addition, it was confirmed from the L-CPL and R-CPL transmission spectra of **Film 1** at *r* = 0, 3, 6, and 9 mm and its reflection images that **Film 1** uniformly reflects both L-CPL and R-CPL in the whole film surface (Figure 4A, insets). The average of 10 reflection wavelengths for L-CPL measured at different positions was found to be 448 nm with a standard deviation of 11.9 nm, while those for R-CPL were 456 nm with a standard deviation of 2.88 nm. Thus, the λ_ref_ values of the L-CPL and R-CPL reflection were almost identical, meaning that there is no color irregularity depending on the measured position. Furthermore, the other CLC films with green and red reflection colors (**Film 2** and **Film 3**) were successfully prepared according to the same fabrication procedure of **Film 1**, except that the concentration of the HPC derivative in the lyotropic CLC mixture was changed to 74.4 wt % for **Film 2** and 72.7 wt % for **Film 3**. The reflection peak shifted to the longer wavelength side with a decrease in the concentration of HPC derivatives arising from the geometric expansion of CLC helical pitch length. The reflection peak wavelengths of **Film 2** and **Film 3** at *r* = 0, 3, 6, and 9 mm were ∼550 and 580 nm, respectively. Moreover, the reflection peaks appeared in both the L-CPL and R-CPL transmission spectra, suggesting that **Film 2** and **Film 3** can also reflect both L-CPL and R-CPL by the shear treatment (Figures 4B and 4C). In this way, we have established a promising methodology to prepare the CLC films with both L-CPL and R-CPL reflection by shear treatment of the lyotropic CLCs of HPC derivative regardless of their reflection peak wavelength.

**Figure 4:**
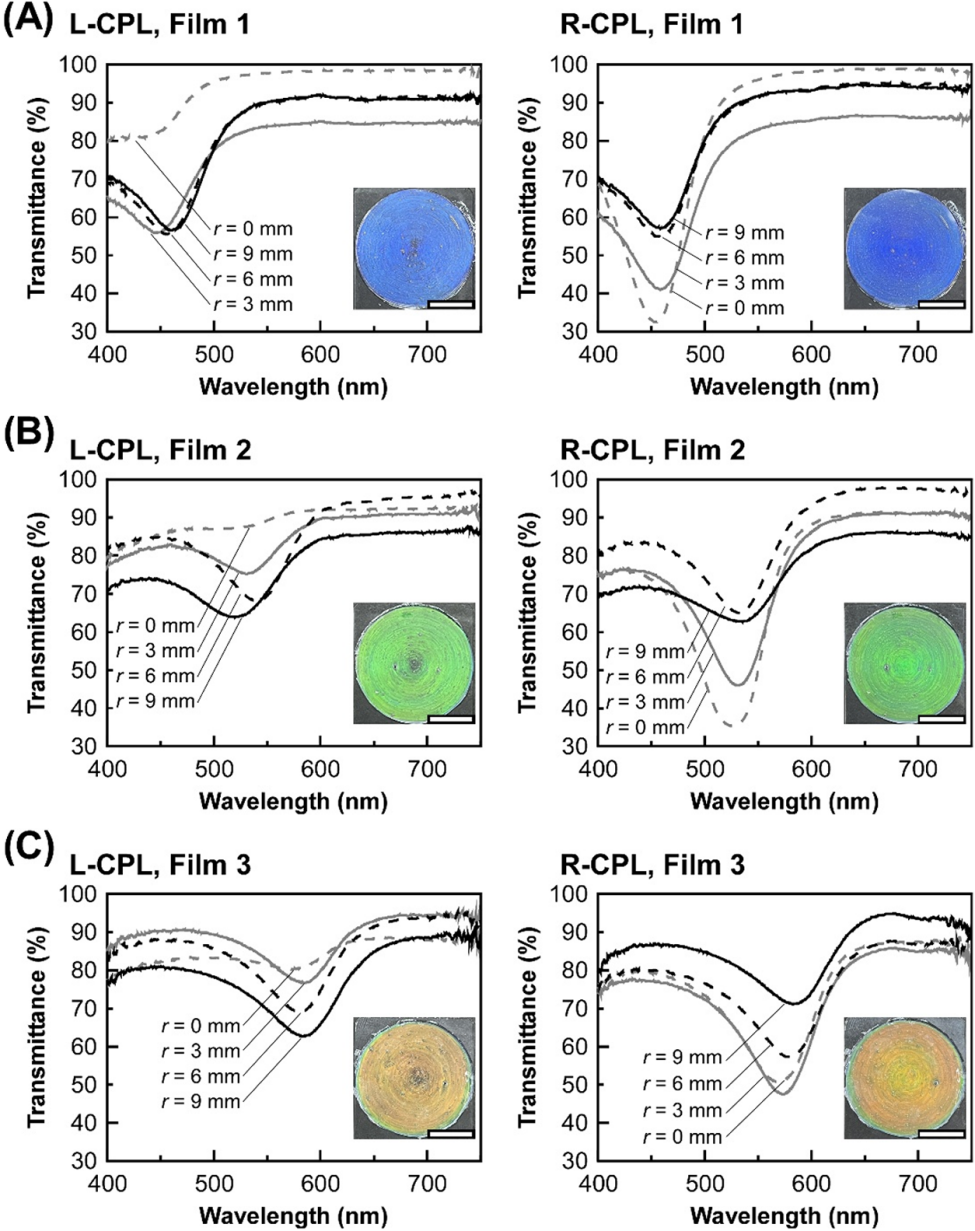
L-CPL (left-side) and R-CPL (right-side) transmission spectra of **Film 1** (A). **Film 2** (B), and **Film 3** (C) at *r* = 0 (dotted dark gray lines), 3 (dark gray lines), 6 (dotted black lines), and 9 mm (black lines). Insets are the reflection images of L-CPL (left panels, insets) and R-CPL (right panels, insets). White scale bars represent 10 mm.

During the course of our systematic experiments, we confirmed the same results of both R-CPL and L-CPL reflection, even though the lyotropic CLCs were sheared in the reverse rotation direction in the rheometer. Therefore, the rotational direction of the rheometer was irrelevant to the CPL reflection properties. In other words, the intrinsic CPL reflection properties of CLC materials were closely related to the helical handedness of the CLC molecular assemblage.

Moreover, the environmental stability of both R-CPL and L-CPL reflection was evaluated for the cross-linked CLC films prepared by shear treatment with the rheometer. First, even though the CLC film was heated at 110 °C, at which an isotropic phase transition was observed for the lyotropic CLC fluid precursor, the transmission spectra of R-CPL and L-CPL were almost unchanged (Supporting Information, Figure S8A). The disappearance of thermotropic CLC behavior is ascribed to the preservation of the CLC structure by the fully cross-linking reaction of the HPC derivative with butyl acrylate. Second, both R-CPL and L-CPL reflection could be maintained even after the CLC film was stored at room temperature for over 2 years (Supporting Information, Figure S8B). Finally, both R-CPL and L-CPL reflection properties of the CLC film showed enough resistance to mechanical compression even after 10 cycles of compression up to 20% strain and release (Supporting Information, Figure S8C). These experimental results imply that such an outstanding characteristic would be greatly advantageous to be able to fabricate the cellulose-refined photonic devices with unique properties of CPL reflection from both technological and sustainable-consumption viewpoints.

As an extension of our findings, we found that the present methodology can be applied to another cellulose derivative. Ethyl cellulose is known to self-organize the CLC phase with intrinsic left-handed helical molecular assemblage in acrylic acid, thereby leading to the selective light reflection of L-CPL. After irradiation with UV light, the CLC solid-state film exhibited L-CPL reflection.^64^ Based on this precedent, we succeeded in the fabrication of the CLC films with both R-CPL and L-CPL reflection by irradiation with UV light after applying the shear treatment to a lyotropic CLC mixture of a cross-linkable ethyl cellulose derivative and acrylic acid (Supporting Information, Figure S9). This result provides conclusive proof that our methodology in this study is universally applicable to other cellulose derivatives or other CLC materials, regardless of the handedness of helical molecular assemblage.

To investigate the effect of film thickness, the CLC films with a thickness of ∼0.3 or 0.8 mm were prepared from the lyotropic CLC mixtures at the polymer concentration of either 74.4 or 71.9 wt % under the same shear conditions, except for the difference in *d*_set_ value (Table 1, **Films 4**–**7**). All of the films reflected both R-CPL and L-CPL, and the reflection peaks of **Films 4**–**7** emerged at ∼549, 539, 609, and 593 nm, respectively. However, the reflection intensity of L-CPL decreased for the CLC films with a thickness of ∼0.3 mm. In fact, even though the peak depth in the transmission spectrum was more than 30% for **Film 4** and **Film 6**, it drastically decreased to ∼10% for **Film 5** and **Film 7** (Supporting Information, Figure S10). Therefore, we investigated the influence of the *d*_set_ value and other conditions to prepare the CLC films in the following section.

### Effect of Shear Treatment Condition on Circularly Polarized Reflection Properties

Both R-CPL and L-CPL reflection phenomena are attributed to the increase in optical retardation (*R*_e_). The CLC films were prepared from the same lyotropic CLC mixture at the polymer concentration of ∼ 77.0 wt % under the same shear conditions, except for the difference in *d*_set_ value (**Films 8**–**13**) to investigate the effect of film thickness on the light reflection properties. Although the *d*_set_ values of **Films 8**–**13** were adjusted to 0.80, 0.50, 0.27, 0.21, 0.15, and 0.10 mm, and their actual film thickness determined by a micrometer caliper (*d*_act._) were 0.86, 0.53, 0.32, 0.29, 0.23, and 0.13 mm, respectively, probably due to the density changes upon cross-linking reaction (Table 1). In the reflection peaks of R-CPL and L-CPL transmission spectra of **Films 8**–**13**, the peak intensity of L-CPL became weaker relative to that of R-CPL as the *d*_set_ value decreased (Supporting Information, Figure S11). This experimental result led to the idea that the reflection intensity of L-CPL might be affected by an increase in *R*_e_. It is generally known that anisotropic mechanical deformation, such as shearing or stretching, gives rise to the molecules to orient in the direction of deformation, resulting in an increase in the *R*_e_ value. Furthermore, the enhancement of the *R*_e_ value affects the polarization state of the incident light because the optical retardation means the difference in optical path length of linearly polarized light incident parallel to the sample’s orthogonal optical axis. The transmission light of the sample is a composite wave of out-of-phase light, which is elliptically polarized light (EPL) or CPL, depending on the magnitude of the *R*_e_ value. Thus, the polarization state is strongly influenced by the *R*_e_ value. The relationship between *R*_e_ and *d*_act._ is shown in eq 8. Because the *R*_e_ value is proportional to *d*_act.,_ the weakening of the reflection intensity of L-CPL is reasonable as the *R*_e_ value decreases with the structural reduction of *d*_act._.

8 $$R_{e}=\Delta n\times d_{\mathrm{act.}}$$

The observation of the transmission light images of **Films 8**–**13** under the crossed-Nicols qualitatively confirmed that **Films 8**–**13** exhibit optical anisotropy, and that *R*_e_ value increases with *d*_act._ (Supporting Information, Figure S12). The brightness of the transmission images improved with increasing *d*_act._ and interference colors were also observed for **Film 8** (*d*_act._ = 0.86 mm) and **Film 9** (*d*_act._ = 0.53 mm). These results imply that *d*_act._ induces an increase in the *R*_e_ value. In addition, the appearance of the Maltese crosses indicated that the optical axis of these films coincided with the shear direction. In other words, in these disc-shaped films, the direction of the optical axis at a certain point coincides with the tangential or diameter direction of the disc.

Quantitative measurements of optical retardation supported that the *R*_e_ value has a significant impact on the CPL reflection properties of CLC films. Figure 5 shows the reflection intensity ratios of L-CPL and R-CPL (*υ*) of **Films 8**–**13** plotted against their *R*_e_ values. Here, the intensity ratio of L-CPL and R-CPL reflection is defined as follows

9 $$\upsilon =\frac{I_{L-\mathrm{CPL}}-I_{R-\mathrm{CPL}}}{I_{L-\mathrm{CPL}}+I_{R-\mathrm{CPL}}}$$

where *I*_L‑CPL_ and *I*_R‑CPL_ are the intensity of the reflection peak in the L-CPL and R-CPL transmission spectra, respectively.^36^ Notice that the value of *R*_e_ of **Films 8**–**13** is affected by the position (*r*) as well as *d*_act._ (Supporting Information, Figure S13). Because the films that were prepared using the parallel plate jig had different orientation states depending on *r* due to the existence of a *γ̇* distribution, as mentioned above. As shown in Figure 5, the value of *υ* increased from −0.5 to 0.0 with the enlargement of *R*_e_ to 300 nm accompanied by the increase in *I*_L‑CPL_. Subsequently, *υ* became constant at ∼0 when the *R*_e_ value exceeded 300 nm.

**Figure 5:**
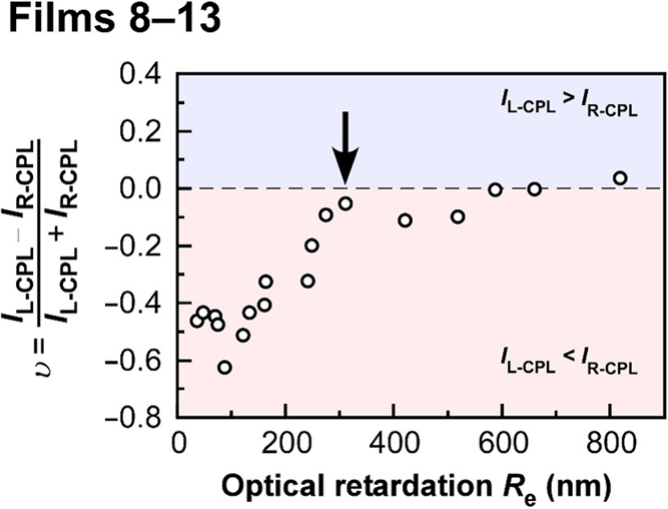
Reflection intensity ratio of L-CPL and R-CPL (υ) dependence on its optical retardation (*R*_e_) of **Films 8**–**13**. *I*_L‑CPL_ and *I*_R‑CPL_ mean the intensity of the reflection peak in L-CPL and R-CPL transmission spectra, respectively. The threshold value of *R*_e_ at which υ = 0, that is, *I*_L‑CPL_ = *I*_R‑CPL_, is *R*_e_ = 300 nm, as indicated by a black arrow.

To prove the versatility of this tendency, we evaluated the relationship between *υ* and *R*_e_ of **Films 4–7**, which were prepared by changing the concentration of butyl acrylate to adjust different wavelengths of the reflection peak. As expected, the value of *υ* increased to ∼0 with a threshold of *R*_e_ at 300 nm in the same manner as the case of **Films 8**–**13** (Supporting Information, Figure S14). Therefore, it was indicated that the reflection of L-CPL is attributed to an increase in the *R*_e_ value of CLC films regardless of their reflection peak wavelength. The mechanism of the emergence of L-CPL reflection will be described later.

Recovering time also affected the CPL reflection properties of the CLC films. For this purpose, three CLC films were prepared by almost the same lyotropic CLCs mixture at the polymer concentration of 77.8–78.2 wt % and the same shear treatment of *γ̇*_out_ = 0.5 s^–1^ and *d*_set_ = 0.50 mm, except for the difference in recovering time (**Films 14**–**16**). The recovering time of **Films 14**–**16** was set at 10, 100, and 1200 s, respectively. As presented in Figure S15A of the Supporting Information, the L-CPL and R-CPL transmission spectra and reflection images implied that the difference in recovering time has an impact on their reflection properties. CPL transmission spectra of **Films 14**–**16** showed that increasing recovering time does not change the reflection peak wavelength of the films at 460 nm, but sharpens the reflection peak width, probably by the formation of well-ordered CLC alignment (Supporting Information, Figure S15A). The narrow peak width corresponds to the emergence of a vivid reflection color. Even when the recovering time was relatively long, as 1200 s, **Film 16** reflects L-CPL with the same intensity as **Films 14** and **15** (Supporting Information, Figure S15A). This is because Δ*n*, which increased with molecular orientation, hardly decreased over time (Supporting Information, Figure S15B).

### Scanning Electron Microscopic Observation of CLC Films from Cross-Section

Many precedents demonstrated that helical pitches of CLCs can be observed by scanning electron microscope (SEM) from the cross-sectional view, whether they are cellulosic or non-cellulosic. Typically, the striped patterns corresponding to CLC helical pitch length, that is, *p* of eq 1, can be observed for the well-ordered CLC systems.^65–69^ From the previous studies, it seems that SEM observation of the striped patterns with *p* in the cellulosic films might not be straightforward compared with that in the petroleum-based CLC films.

In this study, the CLC films of cross-linkable HPC derivative with butyl acrylate were fabricated without and with the shear treatment by the rheometer for the cross-sectional SEM observation (Table 1, **Film 12’**). Before SEM observations, the CLC films were carefully freeze-fractured by immersing them into liquid nitrogen to make the surface of the cross-section as smooth as possible. When the CLC film was prepared without shear treatment, the cross-sectional SEM image showed that the striped patterns appear in a periodic manner, and are evidently aligned in parallel with the film surface for the entire region (Supporting Information, Figure S16A). By SEM image analysis, the geometric *p* value was determined to be ∼349 nm. Therefore, the theoretical reflection peak wavelength of **Film 12’** could be calculated to be ∼513 nm by eq 1, which was comparable to the actual reflection peak wavelength of ∼506 nm (Table 1, **Film 12’**). Therefore, it was confirmed that the cross-sectional SEM observation is a powerful tool to directly observe the microstructure of CLC helical molecular assemblages. However, as the CLC film was prepared by shearing with the rheometer, the striped patterns were observed for the only limited areas of the outermost surface regions of the CLC film from the cross-sectional view (Supporting Information, Figure S16B, yellow circles). In contrast, such striped microstructures were hardly seen around the central region of film thickness (Supporting Information, Figure S16C). Taking the overall results into account, we considered that the CLC assemblages of cross-linkable HPC derivative with butyl acrylate are likely to be well-ordered in the regions near the outermost film surface by the shear treatment.

### Mechanism of Reflection of Both-Handed Circularly Polarized Light

While the CLC molecules are considered to be less-ordered in the films, they maintained the vivid reflection colors even after shear treatment with the rheometer. It suggested that the CLC films also have a helical molecular assemblage. Therefore, it can be inferred that the two types of regions with less-ordered and well-ordered helical molecular assemblages coexist in the thickness direction of CLC film, that is, heterogeneous orientation. Especially in the CLC films fabricated by shear treatment in this study, the gradient distribution of the orientation state is thought to appear in the film thickness direction (Figure 6). Near the surface of the CLC film (Figure 6, top layer and bottom layer regions), the CLC molecules maintain their CLC helical molecular assemblage, probably from the anchoring effect of liquid crystals by the substrate surface of the jig in the rheometer, which contributes to the clear appearance of R-CPL reflection colors. On the other hand, around the middle layer region inside the film (Figure 6, middle layer region), the CLC molecules are less-ordered by the mechanical shear force, which contributes to the increase in the value of *R*_e_.

**Figure 6:**
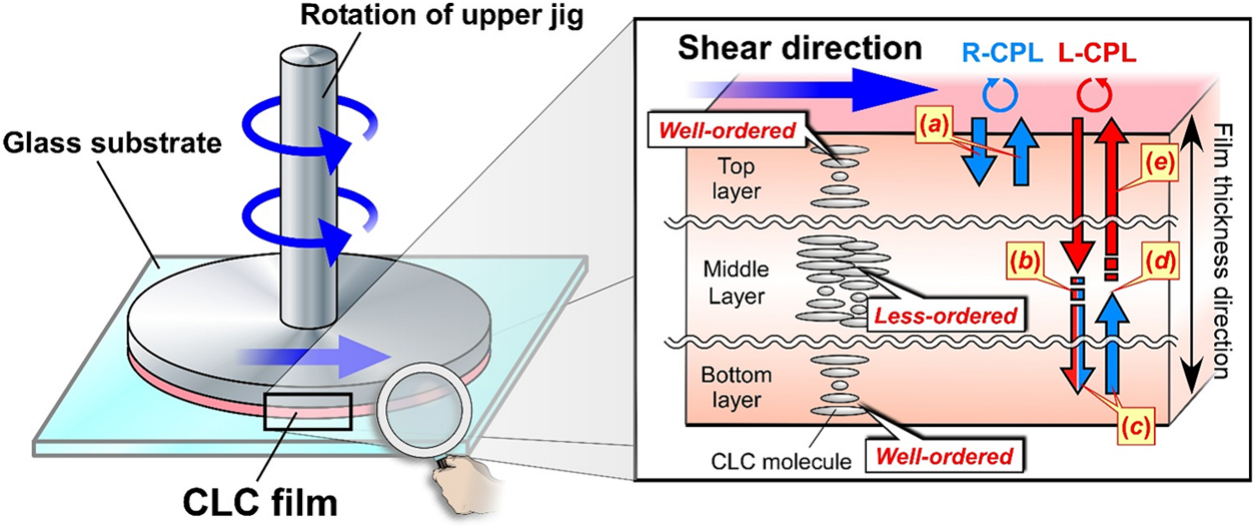
Illustration of the solid-state CLC films of HPC derivative with heterogeneous orientation fabricated by shear treatment with the rheometer. Gradient distribution of CLC molecular orientation state appears in the films by shear treatment, thereby leading to intriguing chiroptical properties of both R-CPL and L-CPL reflection. The well-ordered CLC helical molecular assemblage is maintained in the top and bottom layer regions by the anchoring effect near the jig interface, whereas the molecules are less-ordered around the middle layer region.

As illustrated in Figure 6, the shear treatment produces the gradient orientation distribution of liquid crystal molecules, which is also suggested by the previous report.^70^ Hussain and collaborators successfully fabricated petroleum-based CLC films with no CPL dependence in the reflection light by mechanically stretching the films in a uniaxial direction to increase *R*_e_. According to this previous report, the CLC molecules around the middle layer of the film thickness are disturbed and oriented in the stretch direction, and the CLC molecules around the top and bottom layers form helical molecular assemblage due to the boundary condition. Here, the boundary condition is assumed to refer to molecular motion or relaxation constrained by the entanglement or cross-linking with neighboring CLC molecules. Hussain and collaborators proposed that the distribution of orientation states similar to that in Figure 6 occurs upon the stretching process.

In our previous study, the dynamic viscoelastic measurements of an HPC derivative with a propionyl group also suggested the formation of a strong helical molecular assemblage near the jig surface of the rheometer.^71^ This structure contributes to solid-like behavior, such as the increase and the emergence of a plateau in the storage modulus (*G’*) and the loss modulus (*G*”). However, the region far from the jig surface contributes to the increase in liquidity, which implies that the CLC molecules around the middle layer region can flow easily and are likely to be oriented in the direction of the mechanical shear force. This result supports the existence of the distribution in molecular orientation, as illustrated in Figure 6. Another precedent by Onogi and colleagues on the lyotropic CLCs of an HPC aqueous solution also provided that a strong helical molecular assemblage is formed near the substrate surface of the CLC cell, but the orientation is disordered around its central region of film thickness.^72^ When the shear force was applied to the aqueous HPC solution with a thickness of 90 μm, it formed a helical molecular assemblage with the helical axis vertical to the substrate surface. On the other hand, when the cell thickness was drastically increased 650 μm, such helical molecular assemblage could be formed only in outmost regions up to 120 μm from the substrate surface due to the wall effect.^72^ Very recently, Chazot and colleagues also confirmed that acrylic acid solutions of pristine ethyl cellulose more easily form helical molecular aggregates near the jig surface than in the region far from the jig surface.^73^

When unpolarized light propagates into the CLC film with the orientation distribution depicted in Figure 6, both R-CPL and L-CPL in unpolarized light are reflected by the heterogeneous orientation. The reflection light is considered to be unpolarized light or elliptically polarized light (EPL). When unpolarized light propagates the CLC film, the wavelength component of the incident R-CPL that satisfies the Bragg equation is reflected by the CLC helical molecular assemblage in the top layer region (Figure 6, *a*). On the other hand, that of the L-CPL is transmitted through the top layer and converted to EPL around the middle layer region, whereupon the value of *R*_e_ increased (Figure 6, *b*). Subsequently, the right-handed EPL component is reflected by the CLC helical molecular assemblage of the bottom layer region (Figure 6, *c*) and is converted to EPL again when transmitted through the middle layer region (Figure 6, *d*). Finally, the left-handed EPL component is transmitted through the top layer and is emitted outside the film (Figure 6, *e*). The right-handed EPL is reflected by the CLC helical molecular assemblage in the top layer region, and then converted partially to EPL in the middle layer region, which is thought to pass between the top and bottom layer regions like the optical resonance until it decays. This is how CLC with right-handedness of helical molecular assemblage, which can inherently reflect only R-CPL, reflects both R-CPL and L-CPL induced by shear treatment. Therefore, it would be greatly advantageous to be able to fabricate the high-density sustainable photonic devices of cellulose derivatives only by appropriate shear force, taking the optical retardation into account.

## Conclusions

In this report, we have established a simple strategy to prepare the cellulose-refined CLC films with a unique chiroptical property of both R-CPL and L-CPL reflection phenomenon by shear treatment. In this study, pivotal equipment is the rheometer because it enables us to regulate precisely the shear treatment conditions. When the lyotropic CLC mixtures of the HPC derivative with butyl acrylate were cross-linked for the fabrication of CLC films without the aid of shear treatment, R-CPL reflection dominantly appeared by the intrinsic chirality of the right-handed CLC helical molecular assemblage of the HPC derivative. On the other hand, the CPL reflection property of CLC films could be precisely controlled by the conditions of shear treatment with the rheometer. For the lyotropic CLC mixtures, the shear treatment at *γ̇* = 0.5 s^–1^ enabled the generation of both R-CPL and L-CPL blue, green, and red reflection colors with uniformity of hue in the whole film surface. It was found that such a chiroptical reflection of both R-CPL and L-CPL stems from the molecular orientation in the shear direction, which increases *R*_e_. Rigorous measurements of optical retardation of sheared CLC films with the Sénarmont method revealed that the large *R*_e_ value gives rise to the change in the polarization state of incident light, and the CLC films show both R-CPL and L-CPL reflection with the same intensity at the value of *R*_e_ over 300 nm, regardless of their reflection peak wavelength. Since the CLC film had both vivid reflection color and an increase in optical retardation, it is assumed that regions of well-ordered and less-ordered CLC helical molecular assemblage are gradually separated and coexist in the film thickness direction. The cellulose-refined CLC films with controllability of chiroptical properties would attract a great deal of attention toward the technological development of smarter photonic devices such as 3D displays, information storage and processing, spintronics-based devices, and ellipsometry-based tomography. Moreover, this report provides potential guidelines to produce environmentally and human-friendly photonic devices by refining cellulose for the realization of a sustainable society.

## Supplementary Material



## Notes

Experimental procedures, materials, synthesis and characterization of cross-linkable HPC derivative, preparation of lyotropic CLC mixtures, fabrication of CLC films fabricated by shear treatment, FT-IR and ^1^H NMR spectra, reflection spectral results and their shear treatment effect, illustration of optical systems to measure CPL transmission spectra and optical retardation, cross-sectional SEM images, and viscosity results (PDF)
