# Supplementary material for: Cellulose-Refined Cholesteric Liquid Crystal Films with Both Right- and Left-Handed Circularly Polarized Light Reflection
Source: Biomacromolecules. 2025 Dec 17;27(1):259–71. doi: 10.1021/acs.biomac.5c01286 (PMC12801298; doi:10.1021/acs.biomac.5c01286)
Supplement: Supplementary file 1 [file bm5c01286_si_001.pdf]

Supporting Information for

Cellulose-Refined Cholesteric Liquid Crystal Films with Both Right- and Left-Handed Circularly Polarized Light Reflection

Yu Sotoyama, Yuki Ogiwara, Kazuma Matsumoto, Koya Sunagawa, Naoto Iwata,* and Seiichi Furumi*

Department of Chemistry, Graduate School of Science, Tokyo University of Science,
1-3 Kagurazaka, Shinjuku, Tokyo 162-8601, Japan

ORCID: 0000-0003-3457-6713 (N.I.); 0000-0003-0592-2314 (S.F.)
*E-mail: n-iwata@rs.tus.ac.jp (N.I.); furumi@rs.tus.ac.jp (S.F.)

S1. Experimental Procedures
S1.1. Materials

A pristine hydroxypropyl cellulose (HPC: Fujifilm Wako Pure Chemical Co.; Viscosity of a 2.0 wt% aqueous solution: 2.0–2.9 mPa·s at 20 °C) was used as a starting material for the synthesis of its derivative. Size-exclusion chromatography (SEC) measurement using the polystyrene standards revealed that the number average molecular weight (M_n) and weight average molecular weight (M_w) are 2.30×10^4 and 4.45×10^4, respectively. The average number of hydroxypropyl groups per HPC monomer unit, that is, molar substitution value (*MS*), was determined to be 4.00 by measuring the ^{1}H-NMR spectrum of pristine HPC in $CDCl_3$ according to the previous report.[S1] HPC was dried under vacuum at room temperature for over 24 h before use. Anhydrous acetone (Kanto Chemical Co. Ltd.; 99.5%) was adopted as a solvent in chemical reaction. Acetyl chloride (Tokyo Chemical Industry Co. Ltd.; 98.0%), 2-acryloyloxyethyl isocyanate (Resonac K.K.; 97.0%), butyl acrylate (Tokyo Chemical Industry Co. Ltd.; 99.0%), and 2-hydroxy-2-methylpropiophenone (Tokyo Chemical Industry Co. Ltd.; 96.0%) was used without further purification. Ultrapure water, used as a poor solvent in reprecipitation of the HPC derivative, was prepared using a water purification system (Merck Millipore, Direct-Q UV 5).

S1.2. Synthesis and Characterization of a Cross-Linkable HPC Derivative

In a 200 mL round-bottom flask with nitrogen substitution and shaded with aluminum foil, 6.00 g of dried HPC was completely dissolved in 30.0 mL of anhydrous acetone by stirring at room temperature. After that, 1.29 g of 2-acryloyloxyethyl isocyanate (0.60 eq. to the number of HPC monomer units) was portionwise added into the solution and the reaction proceeded for 20 h at room temperature. Subsequently, 4.65 g of acetyl chloride (3.90 eq.) was added dropwise. After proceeding for additional 24 h, the reaction solution was purified by dropping into ~1 L of ultrapure water to yield a yellow sticky product. This crude product was purified by four cycles of reprecipitation from acetone to ultrapure water. Finally, the purified product was dried in vacuo at room temperature for at least 48 h under dark to obtain the target HPC derivative, which was a clear and slightly yellow polymer melt.

To confirm the reactivity of hydroxy groups of HPC, FT-IR and ^{1}H-NMR spectra were measured for the HPC derivative. FT-IR spectra were acquired using an FT-IR spectrometer (JASCO, FTIR-4700) equipped with an attenuated total reflection (ATR) unit with a diamond prism (JASCO, ATR Pro One), and ^{1}H-NMR spectra in $CDCl_3$ with an internal standard of tetramethylsilane were recorded on an NMR spectrometer (JEOL, ECZ 400). The values of M_n and M_w of pristine HPC were determined by an SEC system (Tosoh, HLC-8220 GPC) combined with a refractive index detector. The SEC measurements were carried out at 40 °C by flowing tetrahydrofuran (Kanto Kagaku Co. Ltd.; 99.5%) as an eluent at the flow rate of 0.35 mL/min. Lastly, the values of M_n and M_w were calculated from the calibration curve of polystyrene standards.

As presented in Figure S1B, the "peak a" at 2.10 ppm in the ^{1}H-NMR spectrum was assigned to the methyl protons of

the acetyl groups, and the value of a stands for the integrated value of “peak a”. The “peak b” at 5.00 ppm in the ^{1}H-NMR spectrum was assigned to the methine protons of the carbamated of acetylated hydroxypropyl groups. Therefore, the value of b is the integration value of peak b at ~5.0 ppm. W is the sum of the integrated values of all peaks derived from protons of the HPC derivative, that is, the sum of the seven hydrogens bonded to the carbon of cellulose, the product of six hydrogens of the hydroxypropyl group. At this time, it was assumed that the hydrogen in the carbamate group is not observed probably because it forms hydrogen bond. The seven hydrogens of 2-acryloyloxyethyl isocyanate correspond to AcC, the three hydrogens of the acetyl group are EtE, and the molar substation degree of pristine HPC is MS. It should be noted that MS is determined to be 4.00 as described in the preceding section.

$$\frac{a}{W} = \frac{3EtE}{7 + 6MS + 7AcC + 3EtE} \tag{5}$$

$$\frac{b}{W} = \frac{AcC + EtE}{7 + 6MS + 7AcC + 3EtE} \tag{6}$$

Dividing Equation (5) by Equation (6) yields the following Equation (S1).

$$\frac{a}{b} = \frac{3EtE}{AcC + EtE} \tag{S1}$$

Solving Equation (S1) for AcC gives the following Equation (S2).

$$AcC = \frac{3b - a}{a} EtE \tag{S2}$$

Substituting Equation (S2) into Equation (2) gives the following Equation (S3).

$$\frac{a}{W} = \frac{3EtE}{7 + 6MS + 7\frac{3b - a}{a} EtE + 3EtE} \tag{S3}$$

Solving Equation (S3) for EtE gives the following Equation (S4).

$$EtE = \frac{a(7 + 6MS)}{3W + 4a - 21b} \tag{S4}$$

Lastly, substituting Equation (S4) into Equation (S2) gives the Equation (S5) for the calculation of AcC.

$$AcC = \frac{(3b - a)(7 + 6MS)}{3W + 4a - 21b} \tag{S5}$$

S1.3. Preparation of Lyotropic Cholesteric Liquid Crystal Mixtures

Lyotropic cholesteric liquid crystal (CLC) mixtures were prepared by dissolving the cross-likable HPC derivative synthesized in the preceding section in a binary mixture of butyl acrylate (Main Text, Figure 1, iv) and 2-hydroxy-2-methylpropiophenone (Main Text, Figure 1, v). At certain concentrations, the reflection color was observed at room temperature. Notice that the reflection peak wavelength of these lyotropic CLCs can be controlled by changing the concentration of the HPC derivative because both butyl acrylate and 2-hydroxy-2-methylpropiophenone are liquid at room temperature and miscible to the cross-likable HPC derivative.[S2] At this time, the concentration of 2-hydroxy-2-methylpropiophenone was standardized at ~0.7 wt%. The HPC derivative was completely dissolved into the mixture by manual stirring with a spatula and by a planetary centrifuge mixer (Thinky, AR-100). Successively, the CLC mixture was homogenized by defoaming with the planetary centrifuge mixer, followed by placing in vacuum for a few min to remove any remaining air bubbles. In this study, the concentration of lyotropic CLC mixture was denoted by the weight concentration of the cross-likable HPC derivative.

S1.4. Fabrication Procedure of CLC Films by Shear Treatment

A stress-controlled rheometer (Anton Paar, MCR 102) was used to perform the shear treatment to the lyotropic CLC mixture. A stainless-steel parallel plate with 25 mm in diameter (Anton Paar, PP25) was used as the upper jig. The temperature of the samples during the shear treatment was maintained at 25 ºC using a Peltier element temperature control system (Anton Paar, P-PTD 200 G/L) equipped with a glass plate with a thickness of ~3.5 mm as a lower jig, which enabled the photo-irradiation with UV light at 365 nm. Therefore, the sheared CLC mixture was irradiated with UV light from the bottom side of rheometer.

First, the lyotropic CLC was set on the rheometer (Main Text, Figure 2, *a*). An appropriate amount of lyotropic CLC was placed on the glass plate of the lower jig. Then the upper jig was lowered to flatten the CLC to set d_{set}, where the value d_{set} means a geometric gap distance between the upper and the lower jigs set by the rheometer, and then the CLC that protruded from the upper jig was removed with a spatula.

After that, the shear treatment was performed by rotating the upper jig at a constant $\dot{\gamma}_{\mathrm{out}}$ (Main Text, Figure 2, *b*). Most of the shear treatments were performed at $\dot{\gamma}_{\mathrm{out}} = 0.5\ \mathrm{s}^{-1}$, and the shear treatment was carried out for 300 s. This time was determined from the apparent viscosity change over time of the lyotropic CLC mixture at the polymer concentration of 77.0 wt% subjected to shear treatment at $\dot{\gamma}_{\mathrm{out}} = 0.5\ \mathrm{s}^{-1}$. The shear treatment was regarded as complete when the apparent viscosity reached a steady state (Supporting Information, Figure S17).

After the shear treatment, the $\dot{\gamma}_{\mathrm{out}}$ was immediately set to 0 s^{-1} (Main Text, Figure 2, *c*) in order to allow to stand the lyotropic CLC. This setting is necessary because the elastic element of the CLC molecules might cause the upper jig to rotate in the opposite direction of the shear direction, thereby reducing the molecular orientation of the shear treatment.

The lyotropic CLC was allowed to be static by keeping it at $\dot{\gamma}_{\mathrm{out}} = 0\ \mathrm{s}^{-1}$. The treatment had the effect of improving the optical reflection property of CLC and making their reflection color more vivid. According to our previous study, immediately after shear flow, the intensity of the reflection peak decreased, and the peak width was broadened in the reflection spectrum.[S3] On the other hand, we also confirmed in the study that light reflection properties are recovered at certain keeping time after the shear flow.

Finally, CLC films were prepared by curing the lyotropic CLC mixtures by initiating cross-linking reaction between HPC derivative and butyl acrylate by irradiation with UV light at 365 nm (Main Text, Figure 2, *d*). As photo-irradiation of the lyotropic CLC with UV light, a light source system of light-emitting diode (LED) (Asahi Spectra, CL-1501) equipped with an LED head unit (Asahi Spectra, CL-H1-365-9-1-B) and wide-angle lens (Asahi Spectra, CL-H1LCB01) was employed in this study. The UV-irradiation was carried out at the intensity of 80 $\mathrm{mW/cm^2}$ for the time of 360 s, corresponding to the UV-irradiation amount of 28.8 $\mathrm{J/cm^2}$. At this time, the UV intensity was measured through a glass plate with a thickness of ~3.5 mm using a photodiode sensor (Ophir, PD300-UV,).

The storage modulus (*G*') and loss modulus (*G*") were monitored by applying oscillatory shear strain at the shear strain of 0.01% and the frequency of 1 Hz, beginning 30 s after the start of UV-irradiation to confirm that *G*' value reaches to a constant value and that the crosslinking reaction is completed. The measurement was not initiated 30 s after the start of UV-irradiation to avoid the oscillatory shear strain affecting the CLC molecular orientation. The custom-built solvent trap for the rheometer was used throughout the process for the preparation of CLC films to minimize the evaporation of solvent.

S1.5. Scanning Electron Microscopic Observation of CLC Films from Cross-Sectional View

Scanning Electron Microscope (SEM) images of CC films from cross-sectional view were taken on a field emission SEM (JEOL, JSM7800FPRIME) operated at an acceleration voltage of 5 kV. Before SEM observation, the CLC films were freeze-fractured by immersing them into liquid nitrogen, and coated with a thin layer of osmium oxide.

S2. Supplementary Figures

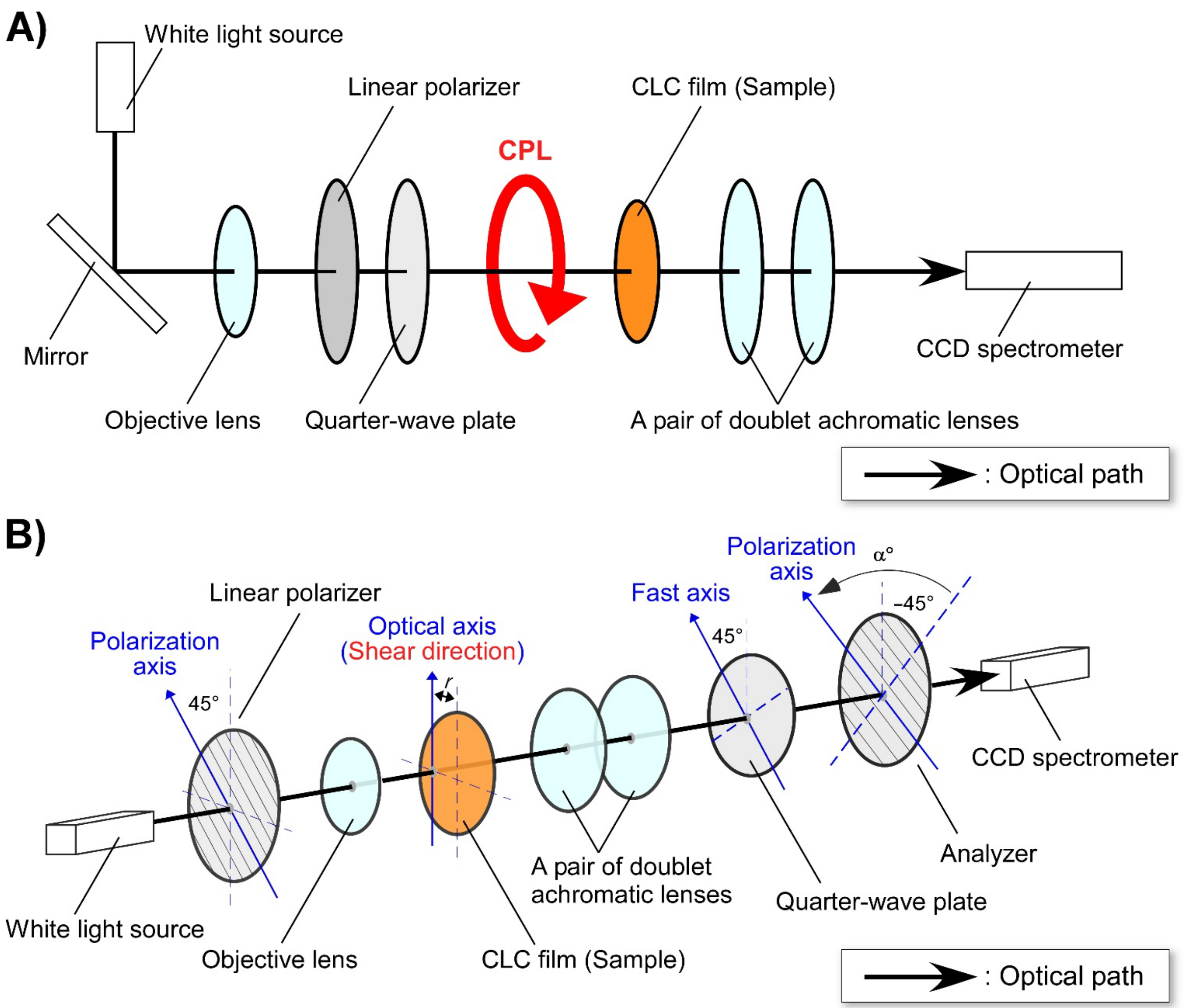

Figure S1. Schematic diagrams of optical measurement systems. (A) Circularly polarized light (CPL) transmission spectral measurement system. (B) Optical retardation (R_e) measurement system by the Sénarmont method.

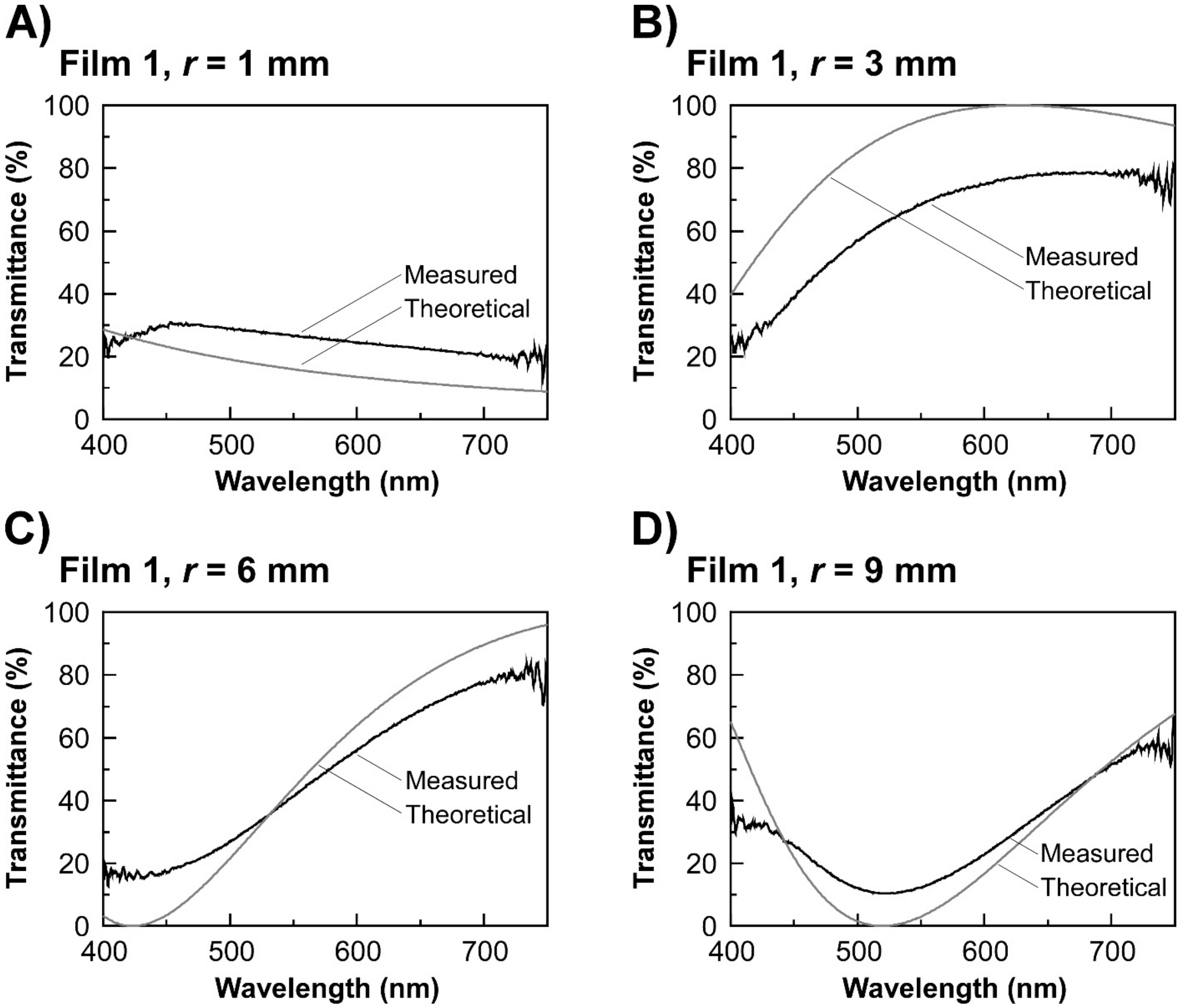

Figure S2. Transmission spectra of **Film 1** under crossed-Nicols at r =1 mm (**A**), 3 mm (**B**), 6 mm (**C**), and 9 mm (**D**). Black lines represent measured spectra and dark gray lines represent the theoretical spectra calculated from R_e calculated by the Sénarmont method.

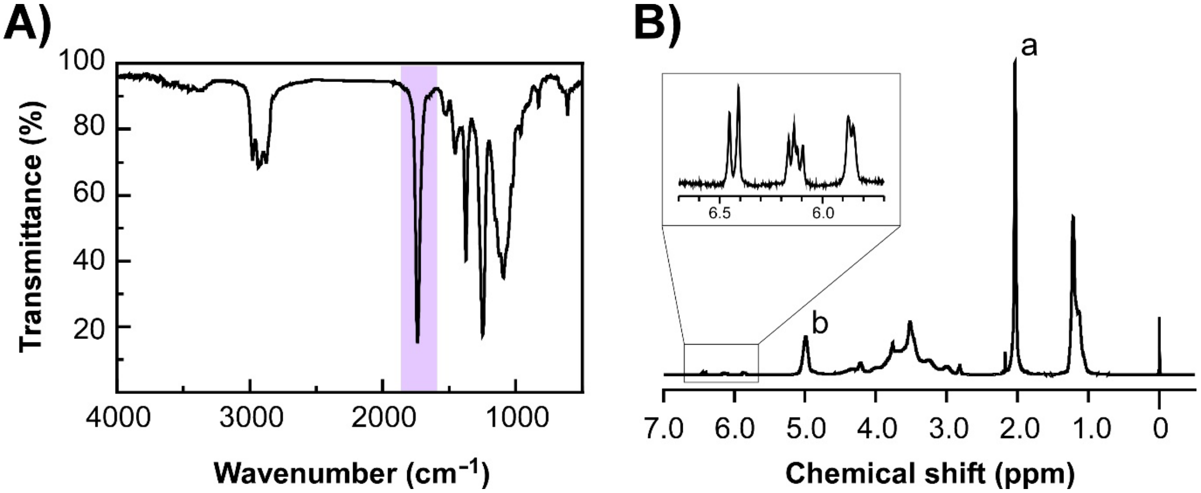

Figure S3. The representative attenuated total reflection (ATR) FT-IR spectrum **(**A) and ^{1}H-NMR spectrum **(**B**)** of the cross-linkable HPC derivative used for fabrication of **Film 1**. In the ATR FT-IR spectra, a peak at 1700 cm^{-1} (indicated purple area) is assigned to C=O stretching vibration. The "peak a" at 2.10 ppm in the ^{1}H-NMR spectrum is assigned to the methyl protons of the acetyl groups. The "peak b" at 5.00 ppm in the ^{1}H-NMR spectrum is assigned to the methine protons of the carbamated of acetylated hydroxypropyl groups. Three peaks appearing in 5.7–6.5 ppm in the ^{1}H-NMR spectrum are assigned to the protons in acryloyl groups.

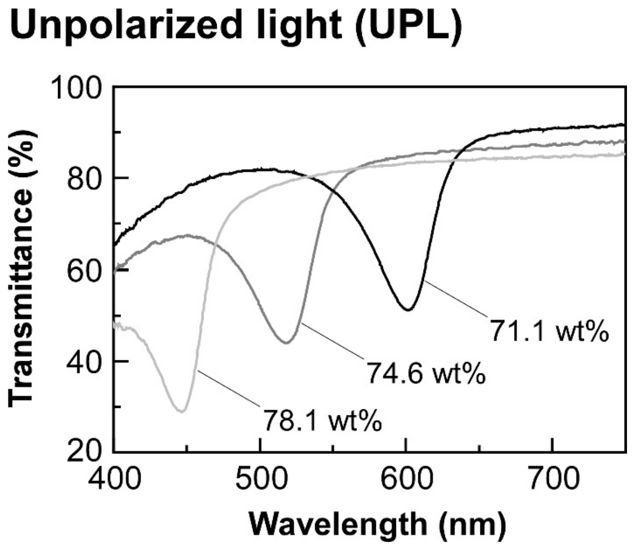

Figure S4. Transmission spectra of a series of lyotropic CLC mixtures of the HPC derivative and butyl acrylate at the concentrations of HPC derivative of 78.1 wt% (light gray line), 74.6 wt% (dark gray line), and 71.1 wt% (black line). The transmission spectra were taken by using unpolarized white light for probing.

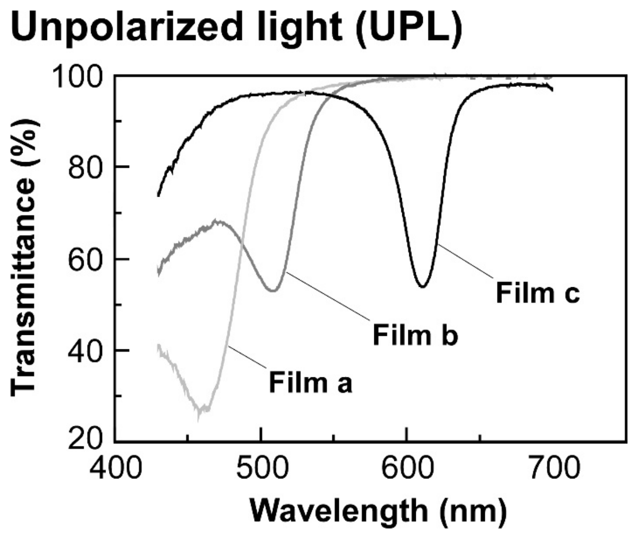

Figure S5. Transmission spectra of **Film a** (light gray line), **Film b** (dark gray line), and **Film c** (black line) by using unpolarized white light for probing.

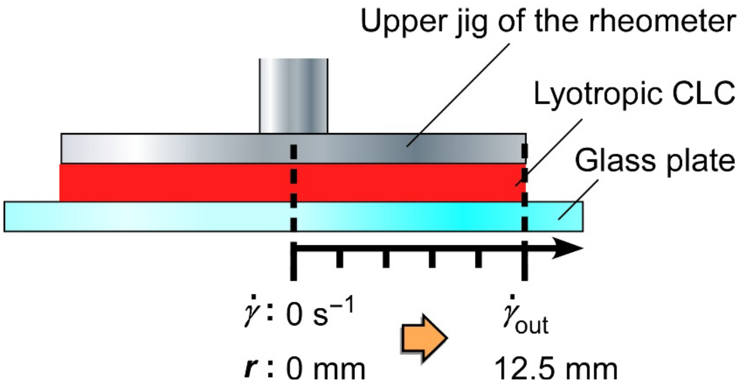

Figure S6. The distribution of shear rate ($\dot{\gamma}$) in a parallel plate jig with 12.5 mm radius of the rheometer. The value of r means the geometric distance between the center and a certain position in a radial direction, and the value of $\dot{\gamma}_{out}$ is the set value of the shear rate at r = 12.5 mm, that is, outermost point of the upper jig. Note that $\dot{\gamma}$ is proportional to r.

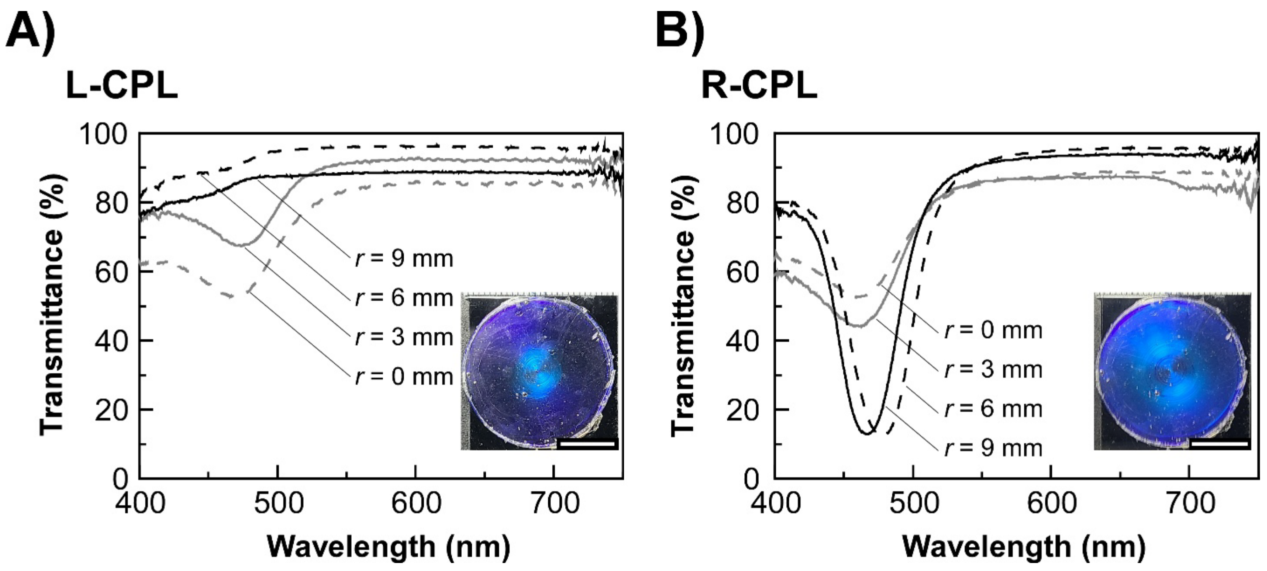

Figure S7. L-CPL (A) and R-CPL (B) transmission spectra of a CLC film fabricated from a mixture of the HPC derivative and butyl acrylate at 78.2 wt% sheared at $\dot{\gamma}_{\mathrm{out}}$ of 5.0 s^{-1} in our preliminary experiment. The spectra were measured at *r* = 0 mm (dotted dark gray lines), 3 mm (dark gray lines), 6 mm (dotted black lines), and 9 mm (black lines). Insets are the reflection images of L-CPL (panel A) and R-CPL (panel B) with white scale bars of 10 mm.

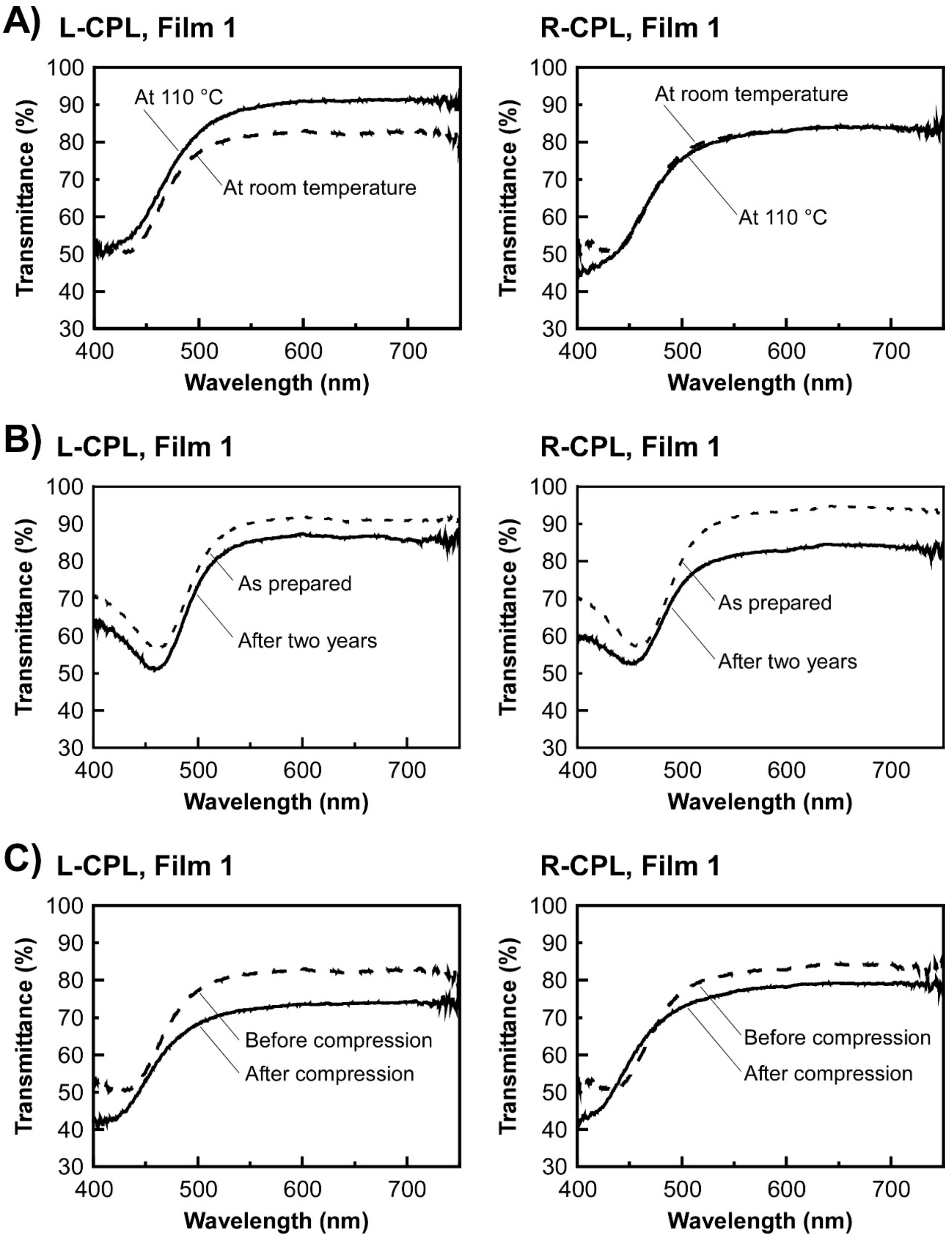

Figure. S8. Experimental results on environmental stability of both R-CPL and L-CPL reflection by **Film 1**. L-CPL transmission spectra (left-hand column) and R-CPL transmission spectra (right-hand column) of **Film 1** before (panels A–C, dotted lines) and after heating at 110 °C (panel A, solid lines), after storing at room temperature over 2 years (panel B, solid lines), and after repeating 10 cycles of compression with 20% strain and release (panel C, solid lines).

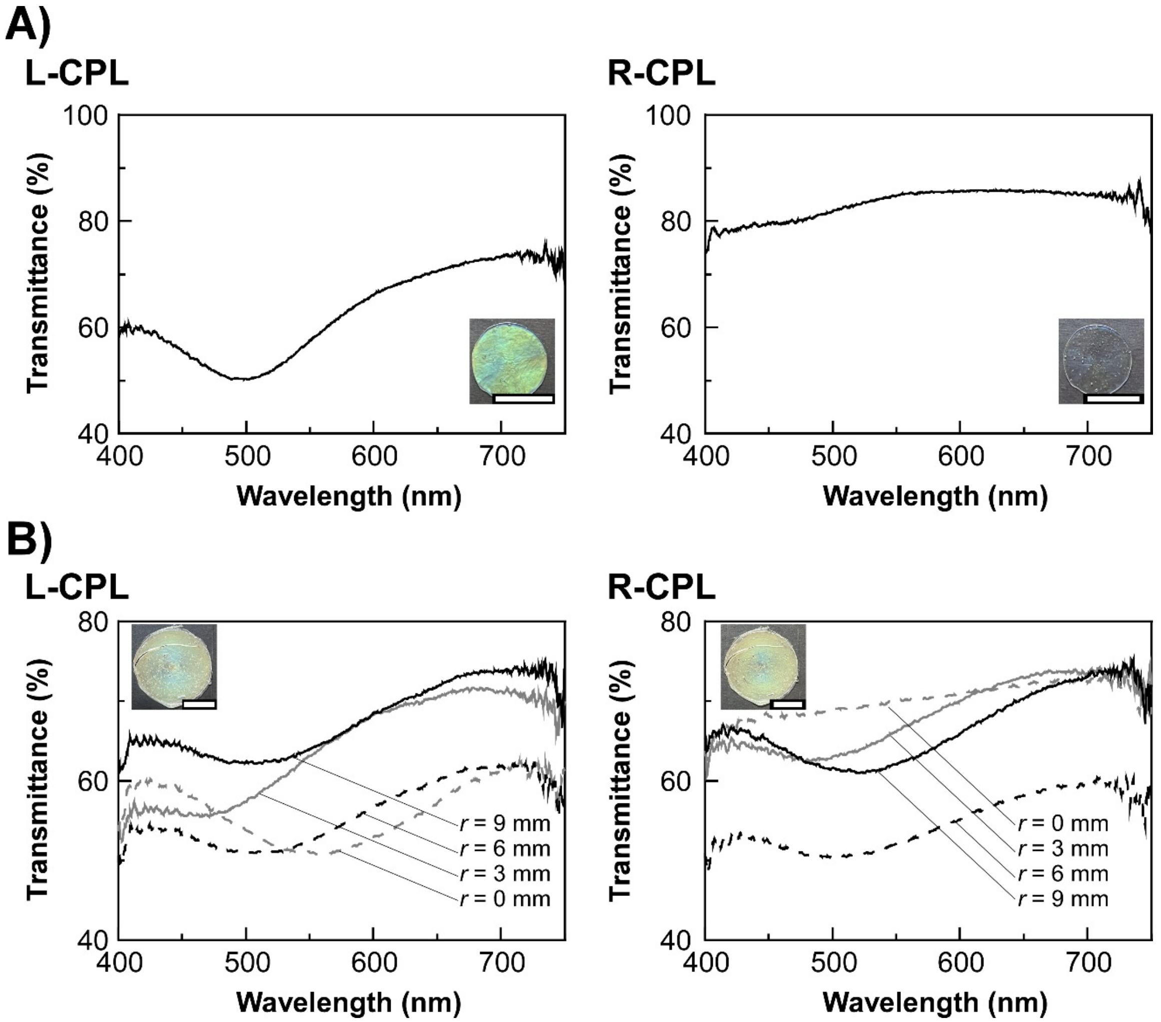

Figure S9. L-CPL (left side) and R-CPL (right side) transmission spectra of CLC films, fabricated from a mixture of a cross-linkable ethyl cellulose derivative and acrylic acid at the polymer concentration of 49.5 wt%, without (A) and with shear treatment with the rheometer (B) at *r* = 0 mm (dotted dark gray lines), 3 mm (dark gray lines), 6 mm (dotted black lines), and 9 mm (black lines). Insets are the reflection images of L-CPL (left side, panels A and B) and R-CPL (right side, panels A and B) with white scale bars of 10 mm. The cross-linkable ethyl cellulose derivative was synthesized by reacting 2-acryloyloxyethyl isocyanate with pristine ethyl cellulose. The shear treatment was carried out at $\dot{\gamma}_{\mathrm{out}} = 0.2\ \mathrm{s}^{-1}$ and recovering time was set at 600 s.

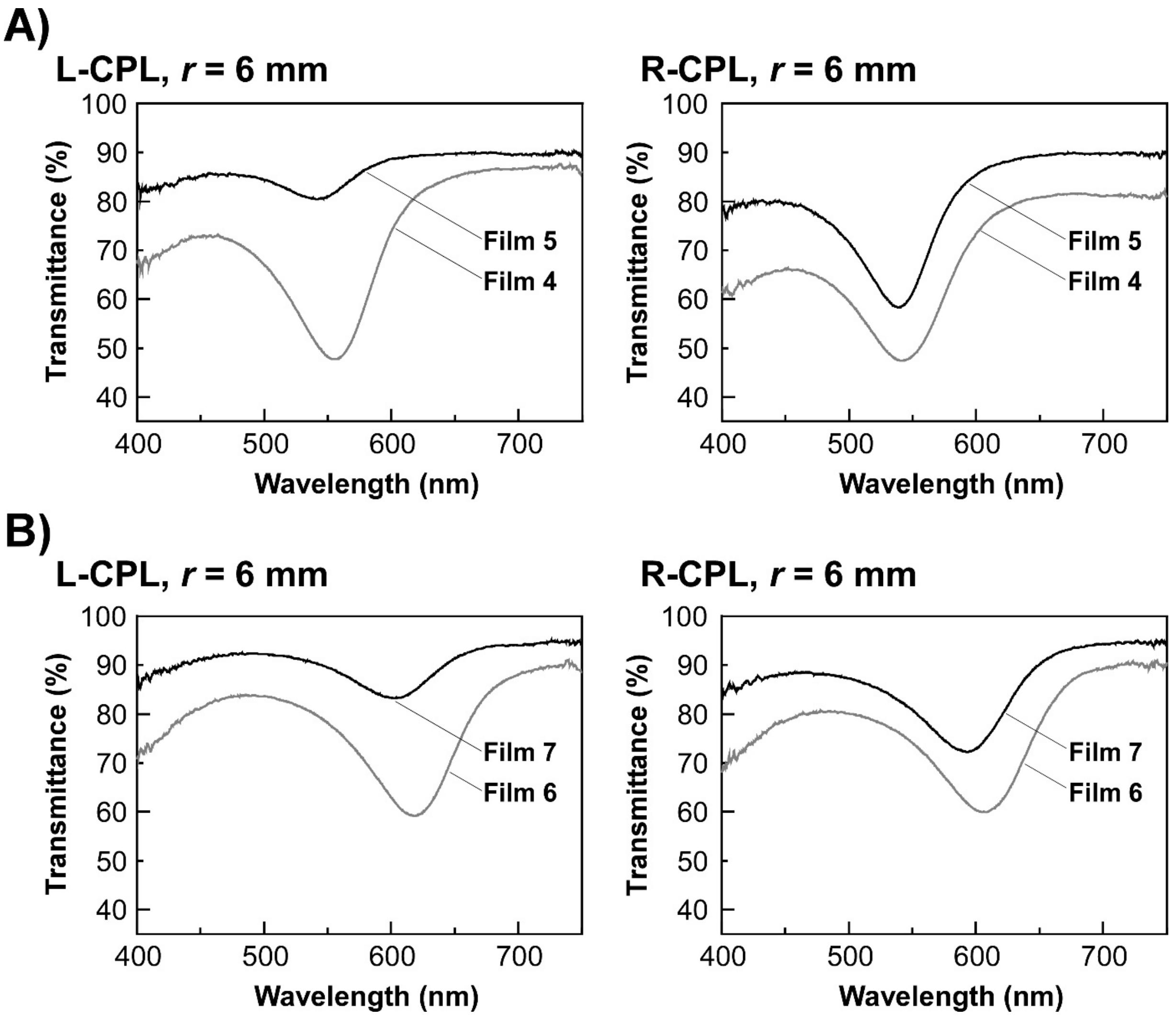

Figure S10. L-CPL (left side) and R-CPL (right side) transmission spectra of **Film 4** (d_{set} = 0.80 mm, dark gray lines) and **Film 5** (d_{set} = 0.30 mm, black lines) fabricated from a mixture of the HPC derivative and butyl acrylate at 74.4 wt% with different values of d_{set} at r = 6 mm (A) and L-CPL (left side) and R-CPL (right side) transmission spectra of **Film 6** (d_{set} = 0.80 mm, dark gray lines) and **Film 7** (d_{set} = 0.30 mm, black lines) fabricated from a mixture of the HPC derivative and butyl acrylate at 71.9 wt% with different values of d_{set} at r = 6 mm (B).

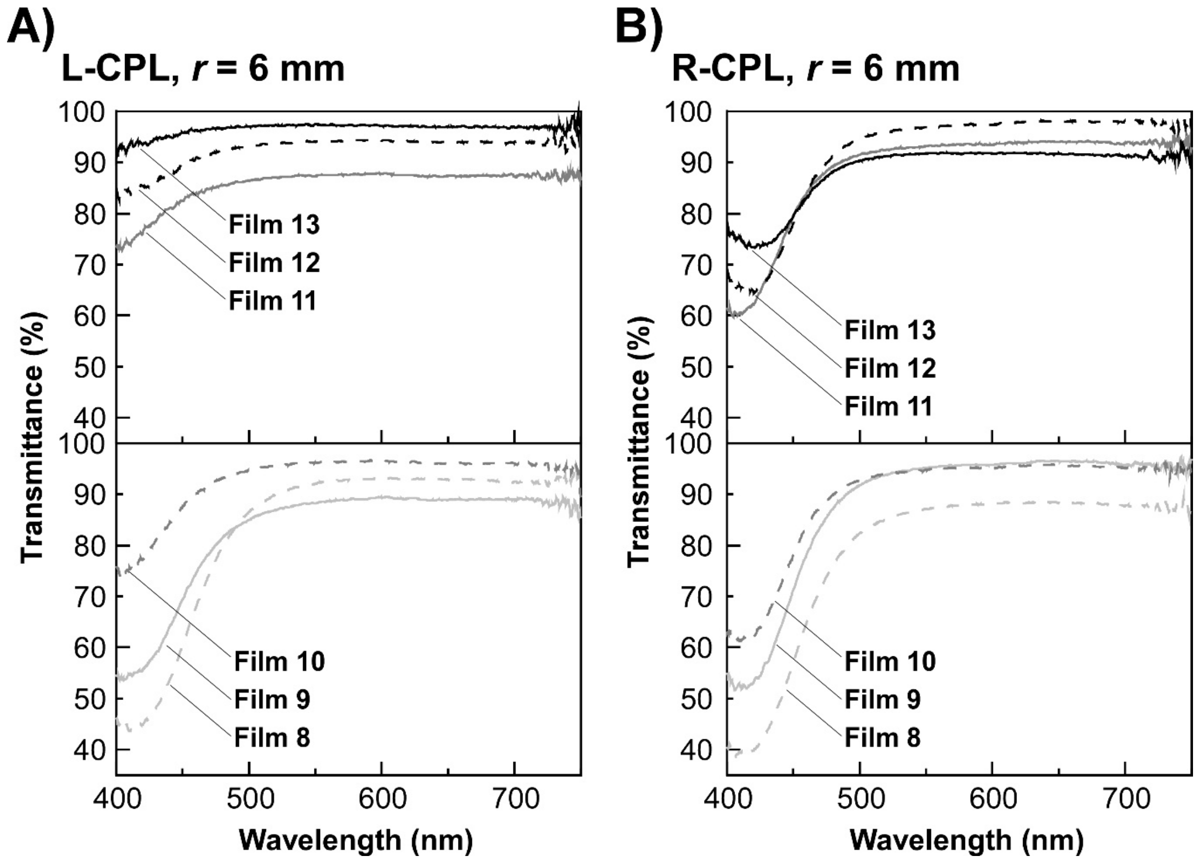

Figure S11. L-CPL (A) and R-CPL (B) transmission spectra of **Film 8** (d_{set} = 0.80 mm, dotted light gray lines), **Film 9** (d_{set} = 0.50 mm, light gray lines), **Film 10** (d_{set} = 0.27 mm, dotted dark gray lines), **Film 11** (d_{set} = 0.21 mm, dark gray lines), **Film 12** (d_{set} = 0.15 mm, dotted black lines), and **Film 13** (d_{set} = 0.10 mm, black lines) fabricated from a mixture of the HPC derivative and butyl acrylate at 77.0 wt%, with different values of d_{set} at r = 6 mm.

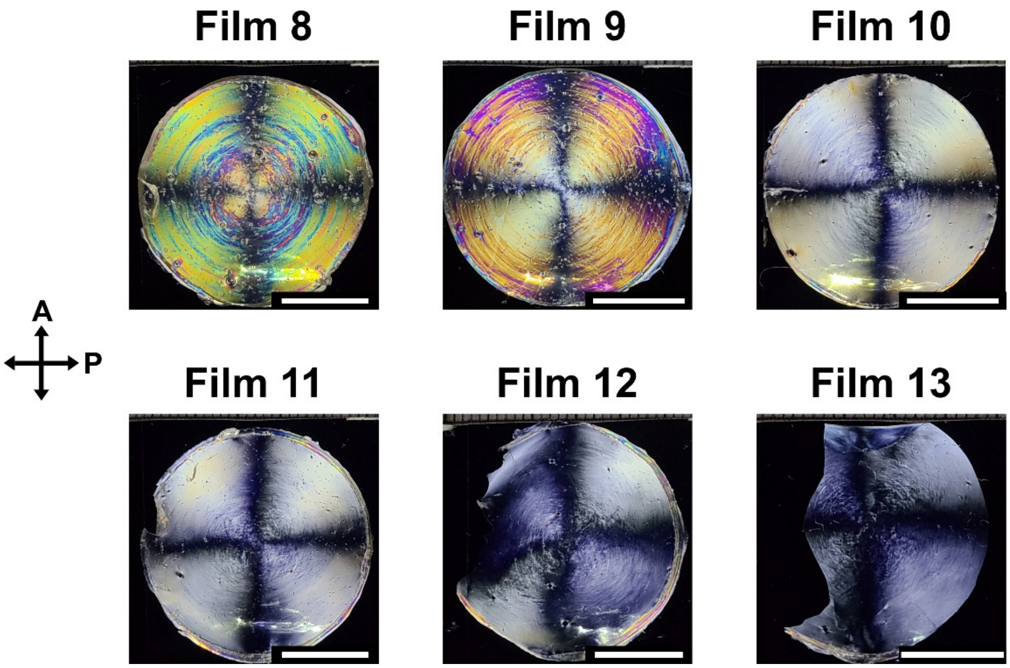

Figure S12. Transmission images of **Films 8–13** (d_{set} = 0.80, 0.50, 0.27, 0.21, 0.15, and 0.10, respectively) fabricated from a mixture of the HPC derivative and butyl acrylate at 77.0 wt% under crossed-Nicols. The directions of A and P mean the directions of the polarization axis of the analyzer and polarizer, respectively. The appearance of Maltese crosses parallel to the polarization axis means that the direction of the optical axis of the films corresponds to the shear direction. **Film 12** and **Film 13** were torn off when it was taken from the upper jig. All white scale bars denote 10 mm.

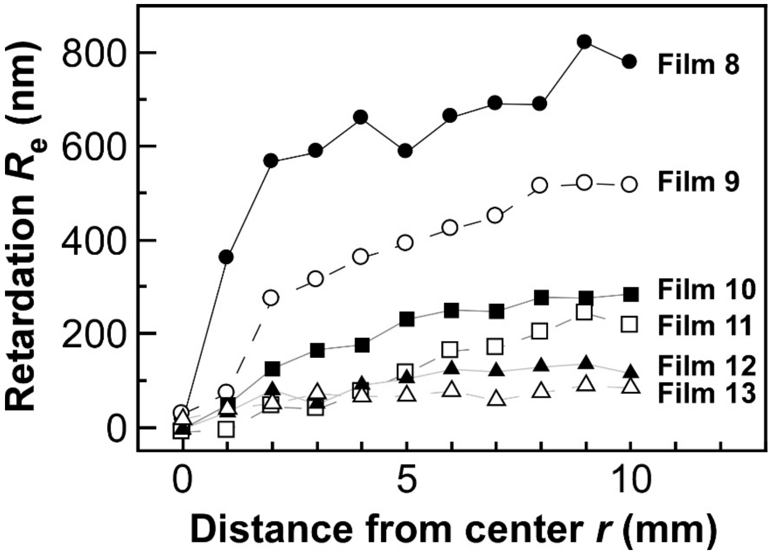

Figure S13. The plot of R_e of **Film 8** (d_{set} = 0.80 mm, closed circles), **Film 9** (d_{set} = 0.50 mm, open circles), **Film 10** (d_{set} = 0.27 mm, closed squares), **Film 11** (d_{set} = 0.21 mm, open squares), **Film 12** (d_{set} = 0.15 mm, closed triangles), and **Film 13** (d_{set} = 0.10 mm, open triangles) fabricated from a mixture of the HPC derivative and butyl acrylate at 77.0 wt% against r.

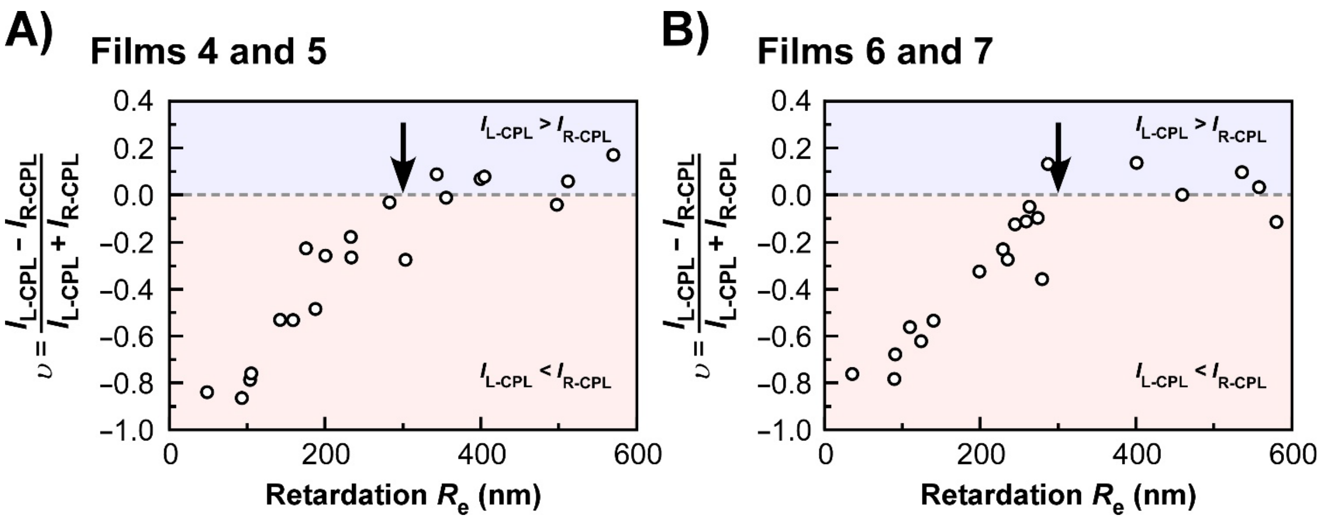

Figure S14. The plot of reflection intensity ratio of L-CPL and R-CPL (υ) against its retardation (R_e) of **Films 4** (d_{set} = 0.80 mm) and **5** (d_{set} = 0.30 mm) fabricated from a mixture of the HPC derivative and butyl acrylate at 74.4 wt% (A) and **Films 6** (d_{set} = 0.80 mm) and **7** (d_{set} = 0.30 mm) fabricated from a mixture of the HPC derivative and butyl acrylate at 71.9 wt% (B). $I_{L\text{-}CPL}$ and $I_{R\text{-}CPL}$ are the intensity of the reflection peak in L-CPL and R-CPL transmission spectra, respectively. The threshold of R_e at which $\upsilon = 0$, that is, $I_{L\text{-}CPL} = I_{R\text{-}CPL}$ is R_e = 300 nm, indicated by black arrows.

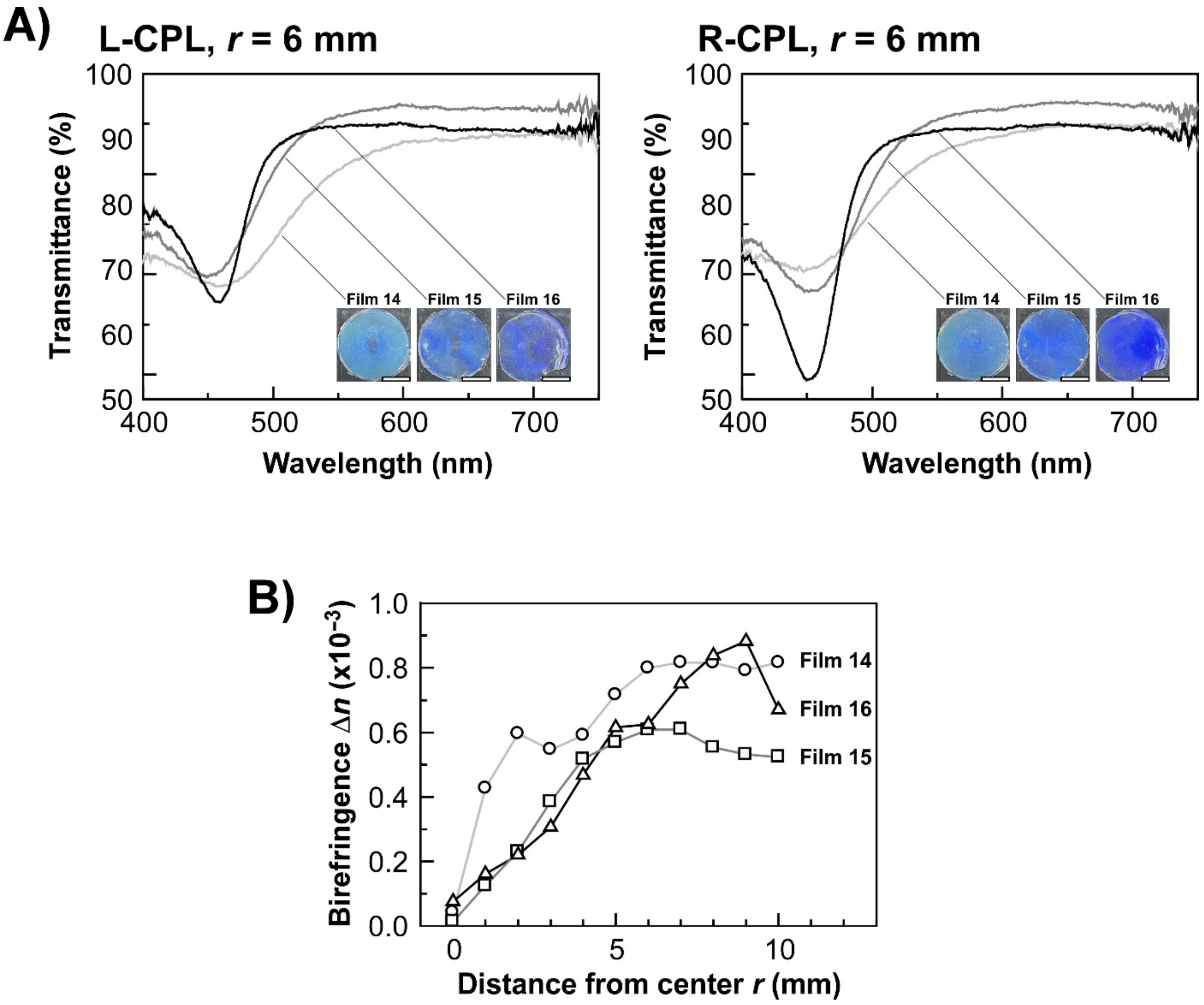

Figure S15. Influence of recovering time on the optical properties of CLC films. (A) L-CPL (left side) and R-CPL (right side) transmission spectra of **Film 14** (10 s, light gray lines), **Film 15** (100 s, dark gray lines), and **Film 16** (1200 s, black lines), at r = 6 mm. The inset images are the reflection images of L-CPL (left side) and R-CPL (right side) with white scale bars of 10 mm. (B) The plot of birefringence (Δn) of **Film 14** (open circles), **Film 15** (open squares) and **Film16** (open triangles) against r.

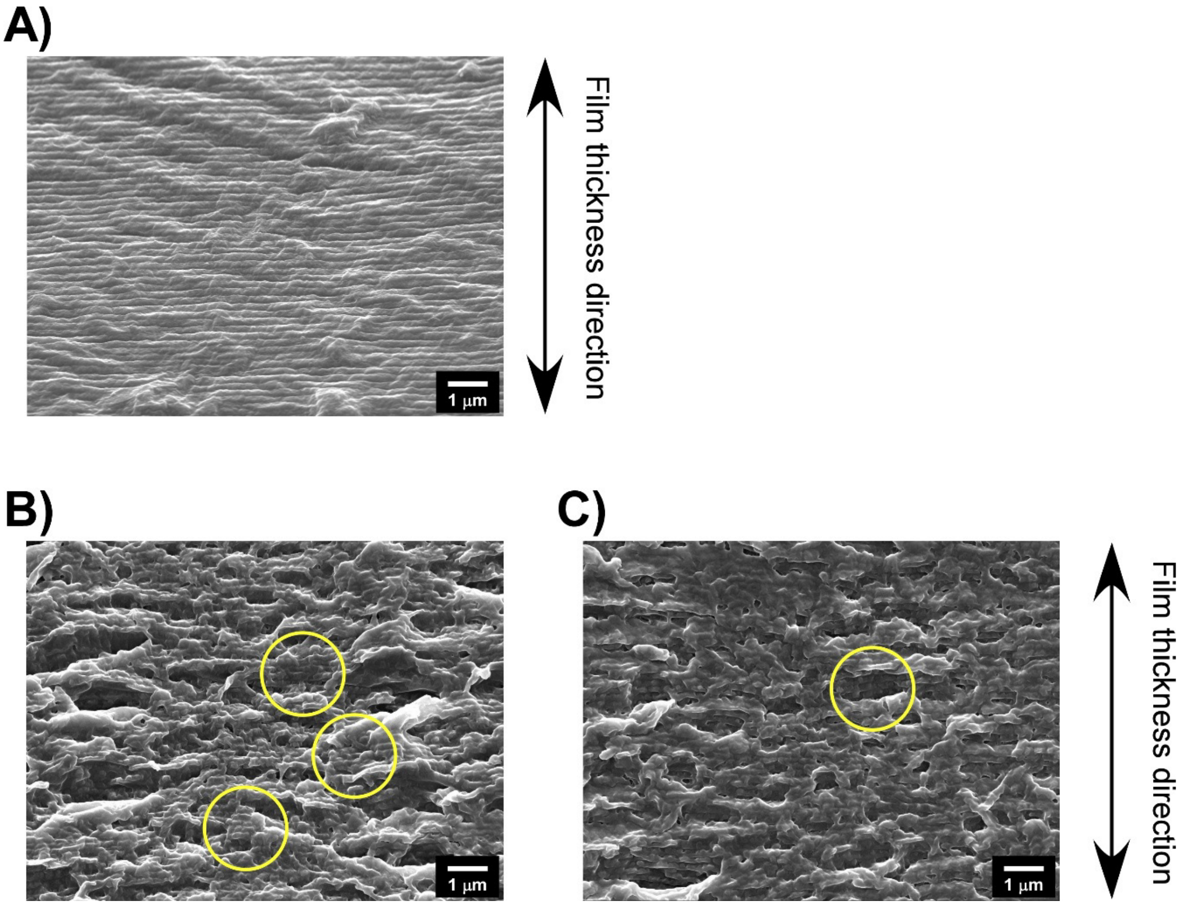

Figure. S16. The cross-sectional SEM images of the cross-linked CLC films prepared without shear treatment (A) and with shear treatment by the rheometer (B and C). When the CLC film was fabricated by shearing with the rheometer, the cross-sectional SEM images of the CLC films were observed near the outermost surface regions (B) and around the central region of film thickness (C). All white scale bars denote 1 μm.

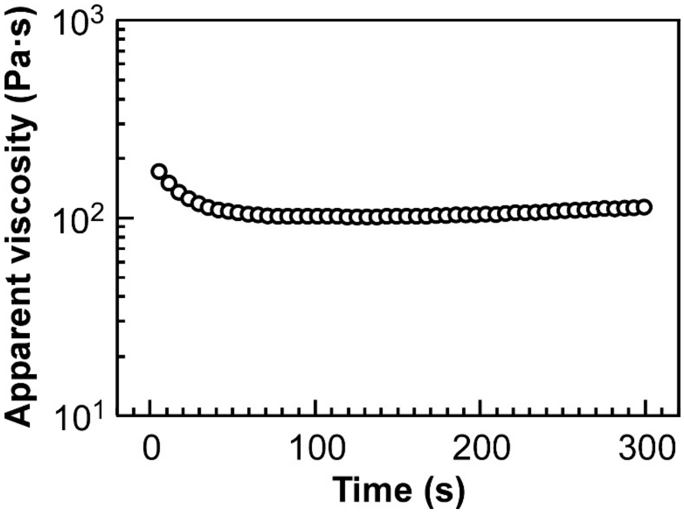

Figure S17. The time dependence of apparent viscosity at $\dot{\gamma}_{\mathrm{set}} = 0.5\ \mathrm{s}^{-1}$, d_{set} = 0.50 mm, and 25 ºC of a lyotropic CLC at the concentration of HPC derivative of 77.0 wt%, where d_{set} means the geometric distance between the upper and lower jigs. This change in viscosity was monitored during the shear orientation treatment when **Film 1** was fabricated.

S3. References

(S1) Ho, F. F. L.; Kohler, R. R.; Ward, G. A. Determination of Molar Substitution and Degree of Substitution of Hydroxypropyl Cellulose by Nuclear Magnetic Resonance Spectrometry. *Anal. Chem.* **1972**, *44* (1), 178–181.

(S2) Fukawa, M.; Suzuki, K.; Furumi, S. Disappearance of Reflection Color by Photopolymerization of Lyotropic Cholesteric Liquid Crystals from Cellulose Derivatives. *J. Photopolym. Sci. Technol.* **2018**, *31* (4), 563–567.

(S3) Ogiwara, Y.; Iwata, N.; Furumi, S. Dominant Factors Affecting Rheological Properties of Cellulose Derivatives Forming Thermotropic Cholesteric Liquid Crystals with Visible Reflection. *Int. J. Mol. Sci.* **2023**, *24* (5), 4269.
